# Identification of *Giardia lamblia* DHHC Proteins and the Role of Protein S-palmitoylation in the Encystation Process

**DOI:** 10.1371/journal.pntd.0002997

**Published:** 2014-07-24

**Authors:** María C. Merino, Nahuel Zamponi, Cecilia V. Vranych, María C. Touz, Andrea S. Rópolo

**Affiliations:** Instituto de Investigación Médica Mercedes y Martín Ferreyra, INIMEC – Consejo Nacional de Investigaciones Científicas y Técnicas (CONICET), Universidad Nacional de Córdoba, Córdoba, Argentina; Georgetown University, United States of America

## Abstract

Protein S-palmitoylation, a hydrophobic post-translational modification, is performed by protein acyltransferases that have a common DHHC Cys-rich domain (DHHC proteins), and provides a regulatory switch for protein membrane association. In this work, we analyzed the presence of DHHC proteins in the protozoa parasite *Giardia lamblia* and the function of the reversible S-palmitoylation of proteins during parasite differentiation into cyst. Two specific events were observed: encysting cells displayed a larger amount of palmitoylated proteins, and parasites treated with palmitoylation inhibitors produced a reduced number of mature cysts. With bioinformatics tools, we found nine DHHC proteins, potential protein acyltransferases, in the *Giardia* proteome. These proteins displayed a conserved structure when compared to different organisms and are distributed in different monophyletic clades. Although all *Giardia* DHHC proteins were found to be present in trophozoites and encysting cells, these proteins showed a different intracellular localization in trophozoites and seemed to be differently involved in the encystation process when they were overexpressed. *dhhc* transgenic parasites showed a different pattern of cyst wall protein expression and yielded different amounts of mature cysts when they were induced to encyst. Our findings disclosed some important issues regarding the role of DHHC proteins and palmitoylation during *Giardia* encystation.

## Author Summary

Giardiasis is a major cause of non-viral/non-bacterial diarrheal disease worldwide and has been included within the WHO Neglected Disease Initiative since 2004. Infection begins with the ingestion of *Giardia lamblia* in cyst form, which, after exposure to gastric acid in the host stomach and proteases in the duodenum, gives rise to trophozoites. The inverse process is called encystation and begins when the trophozoites migrate to the lower part of the small intestine where they receive signals that trigger synthesis of the components of the cyst wall. The cyst form enables the parasite to survive in the environment, infect a new host and evade the immune response. In this work, we explored the role of protein S-palmitoylation, a unique reversible post-translational modification, during *Giardia* encystation, because *de novo* generation of endomembrane compartments, protein sorting and vesicle fusion occur in this process. Our findings may contribute to the design of therapeutic agents against this important human pathogen.

## Introduction

The flagellated protozoan parasite *Giardia lamblia* is a major cause of non-viral/non-bacterial diarrheal disease worldwide. This parasite can cause asymptomatic colonization or acute or chronic diarrheal illness and malabsorption [1]. Infection begins with the ingestion of *Giardia* in its cyst form which, after exposure to gastric acid in the host stomach and proteases in the duodenum, gives rise to trophozoites. The inverse process is called encystation and begins when the trophozoites migrate to the lower part of the small intestine where they receive signals that trigger synthesis of the components of the cyst wall. The encystation process is tightly regulated but the exact mechanism that controls this process is still obscure. Expression of the three Cyst Wall Proteins (CWP) and the glycopolymer biosynthetic enzymes, is largely upregulated. In addition, several other proteins, whose roles in encystation are yet to be discovered, are upregulated at the transcriptional level [2], [3]. Various protein posttranslational modifications (PTM) have been implicated in the development of encystation, such as phosphorylation [4] and deacetylation [5], among others [6], [7], [8]. There is also some evidence of the role of PTM in gene regulation for the control of this process [9].

Protein S-palmitoylation (hereafter referred to as palmitoylation), the post-translational addition of palmitic acid (16∶0) to cysteine residues of proteins, is a PTM essential for proper membrane trafficking to defined intracellular membranes or membrane sub-domains, protein stability, protein turnover, and vesicle fusion [10], [11], [12]. Unlike the other lipid modifications, palmitoylation is potentially reversible, providing a regulatory switch for membrane association [13], [14]. Palmitoylation is catalyzed by a family of protein acyltransferases (PATs), which transfer a palmitoyl moiety derived from palmitoyl-CoA to a free thiol of a substrate protein to create a labile thioester linkage [15], [16]. The discovery of these enzymes came through studies in yeast that identified the PATs Erf2 and Akr1, which are active against Ras and casein kinase, respectively [17], [16]. These enzymes are polytopic integral membrane proteins which share the conserved Asp-His-His-Cys (DHHC) - cysteine-rich domain (CRD). The general membrane topology predictions indicate that the core structure of a PAT is four transmembrane domains (TMDs), with the N- and C- terminus in the cytoplasm [18]. The signature feature DHHC-CRD, which is indispensable for palmitoylating activity, is located in the cytoplasmic loop between the second and third TMDs [19]. There is a small group of PATs that display six TMDs with an extended N-terminal region encoding ankyrin repeats. The yeast PAT called Akr1 is a member of this group [16], [20]. All these findings were crucial in defining palmitoylation as an enzymatic process and led to subsequent identification of protein acyltransferases in many other organisms, such as mammals [21], [22], plants [23], and protozoan parasites like *Toxoplasma gondii* [24], [25], *Plasmodium* [26], [25], and *Trypanosoma brucei* [27].

There is scarce knowledge about palmitoylation in *Giardia*, but some findings indicate that this PTM may play an important role in pathogenesis. It was shown that α19-giardin, one of the major protein components of the *Giardia* cytoskeleton, can be both myristoylated and palmitoylated [28] and that the variant-specific surface proteins (VSPs) may be palmitoylated within their C-terminal domains [29], [30]. Later, Touz et al. determined the exact site of palmitoylation of the VSPs, characterized the enzyme responsible for this modification, and determined the participation of palmitoylation during antigenic variation [31], a process in which the trophozoite continuously changes its surface antigen coat [32]. Antigenic variation and encystation are two distinctive mechanisms of defense that the parasite has developed to survive in hostile environmental conditions during its life cycle, and it has been suggested that both are mechanistically related processes [33].

Accumulation of material in membrane vesicles followed by transport and vesicle fusion and secretion are some of the main events involved in *Giardia* encystation. Because palmitoylation has been reported to play a key role in these events in other cell types [12], [10], [34], [35], [36], it is likely that this PTM may also play a role in *Giardia* encystation. In this work, we address the question of whether PATs and palmitoylation itself are involved in *Giardia* encystation. We provide evidence about the role of palmitoylation in *Giardia* encystation biology by inhibiting this PTM with 2-bromopalmitate (2-BP) or 2-fluoropalmitate (2-FP). Using bioinformatics, we identified the potential PATs (hereafter called DHHC proteins) in the *Giardia lamblia* proteome and performed a phylogenetic analysis of these proteins. We evaluated the expression of the total collection of DHHC proteins in trophozoites and encysting parasites. Using *dhhc* transgenic *Giardia* parasites, we revealed the intracellular localization of DHHC proteins and their influence in CWP expression and cyst yield when parasites were induced to encyst. Our data suggest a role of palmitoylation and DHHC proteins in encystation, providing an insight into the impact of this PTM in *Giardia* survival.

## Methods

### *Giardia lamblia* culture, transfection, and differentiation

Trophozoites of the isolate WB, clone 1267 [37], were cultured in TYI-S-33 medium supplemented with 10% adult bovine serum and 0.5 mg ml^−1^ bovine bile (Sigma, St. Louis, MO) as described [38]. GL50806_40376 (High Cysteine Non-variant Cyst protein; HCNCp), GL50803_1908, GL50803_2116, GL50803_16928, and GL50803_8711 open reading frames (ORF) were amplified from genomic DNA. GL50806_40376 was cloned into the vector **p**TubV5-pac [39] to generate **p**HCNCp-V5 plasmid. GL50803_1908, GL50803_2116, GL50803_16928, and GL50803_8711 were each one cloned into the vector pTubHA-pac [39] to generate the pDHHC-HA plasmids. Trophozoites were transfected with the constructs by electroporation and selected by puromycin (Invivogen, San Diego, CA) as previously described [40], [41], [42]. Trophozoites transfected with empty **p**TubHA-pac or **p**TubV5-pac plasmids were used as control. Primer sequences used for DHHC proteins cloning are depicted in table S1. Encystation was induced by growing trophozoites for one culture cycle in TYI-S-33 medium without bile (pre-encystation). Bile-deficient medium was poured off along with unattached trophozoites and replaced with warmed encysting medium containing 0.45 mg ml^−1^ porcine bile (Sigma, St. Louis, MO) and 0.25 mg ml^−1^ lactic acid (Sigma, St. Louis, MO), pH 7.8, and incubated at 37°C for 48 h [43]. Total encysting cultures were harvested at 48 h by chilling and centrifugation, and subsequently used for palmitoylation assay, RNA extraction, western blot, immunofluorescence, or flow cytometry.

### Palmitoylation assay

The assay followed the procedure described by Papanastasiou *et al.* and Corvi *et al.* [29], [44]. Briefly, 8×10^6^ growing and encysting wild-type or *dhhc* transgenic parasites were washed, suspended in 1 ml of RPMI (Gibco, Invitrogen, Carlsbad, CA) containing 200 µCi of [9,10-^3^H(N)]-palmitic acid (Perkin-Elmer, MA), previously conjugated to BSA fatty acid free (1∶1, mol∶mol ratio), and incubated for 4 h at 37°C. The samples were then suspended on SDS–PAGE loading buffer without any reducing agent and loaded onto SDS-PAGE gel. The gel was then incubated for 30 min in ddH_2_O and for 30 min more in 1M sodium salicylate pH 6.5. The gel was then incubated with 3% glycerol, 10% acetic acid, and 40% methanol for 30 min, dried for 2 h at 80°C using a gel dryer machine, and exposed to autoradiographic film for a month. For hydroxylamine treatment, the gel was soaked in either 1 M NH_2_OH- NaOH pH 7.0 or 1 M Tris-HCl pH 7.0 (Control) for 48 h. Finally, the gel was incubated for 30 min in ddH_2_O and for 30 min more in 1M sodium salicylate pH 6.5, dried as described above, and exposed to autoradiographic film for a month.

### Acyl-biotin exchange

Total cellular palmitoylated proteins from growing and encysting wild-type or transgenic (overexpressing HCNCp) parasites, were purified following the procedure described by Wan *et al.* [45]. Briefly, 5×10^7^ trophozoites or 48 h encysting parasites were harvested and lysed with Lysis buffer (LB; 50 mM Tris-HCl pH 7.4, 5 mM EDTA, 150 mM NaCl) with 10 mM N-Ethylmaleimide (NEM; Thermo Scientific Pierce Rockford, IL) plus protease inhibitors. After sonication, 1.7% of Triton X-100 was added to each sample and incubated for 1 h at 4°C under shacking. The samples were then centrifuged at 500× g for 5 min at 4°C. The supernatant was collected in a new tube and solubilized proteins were precipitated with chloroform-methanol. Proteins were resolubilized in 4% SDS buffer (SB; 4% SDS, 50 mM Tris-HCl pH 7.4, 5 mM EDTA) with 10 mM NEM by incubating at 37°C under shacking. Each sample was then diluted with 3 vol of LB with 1 mM NEM, protease inhibitors, and 0.2% Triton X-100 and incubated overnight at 4°C under shacking. Proteins were then precipitated by three sequential chloroform-methanol extractions after which each sample was dissolved in SB and split into two equal fractions: one for neutral pH hydroxylamine treatment (hyd+) and the other for neutral pH Tris buffer treatment (hyd−). The hyd+ portion was diluted with 4 vol of hyd+ buffer (1M hydroxylamine pH 7.4, 150 mM NaCl, 1 mM HPDP-Biotin, 0.2% Triton X-100, protease inhibitors), and the hyd- portion with 4 vol of the hyd- buffer (50 mM Tris-HCl pH 7.4, 5 mM EDTA, 150 mM NaCl, 1 mM HPDP-Biotin (Thermo Scientific Pierce, Rockford, IL), 0.2% Triton-X-100, protease inhibitors) and incubated for 1 h at room temperature under shacking, followed by chloroform-methanol precipitation. The samples were then resuspended in SB at 37°C under shacking. Protein pellets were solubilized in LB containing 0.2% Triton X-100. Streptavidin-agarose (Thermo Scientific Pierce, Rockford, IL) was added at concentration of 25 µl beads ml^−1^ and the lysate and samples were incubated for 1 h at room temperature. Unbound proteins were removed by four sequential washes with LB containing 0.2% Triton X-100. Samples were finally eluted with 100 mM DTT containing 0.2% Triton X-100. Each eluate was then analyzed by Western blotting.

### Inhibition of palmitoylation

*Giardia* trophozoites were cultured as described above. 2-bromopalmitate (2-BP) (Sigma-Aldrich, St. Louis, MO) or 2-fluoropalmitate (2-FP) (Cayman Chemical, Ann Arbor, MI) were added to the media for 48 h to reach a final concentration of 10, 20, 40, 50, 75 or 100 µM for 2-BP, and 100, 150 or 200 µM for 2-FP. The inhibitors were diluted in DMSO (Sigma-Aldrich, St. Louis, MO) following manufacturer indications. The parasites were then analyzed by staining them with Trypan blue to distinguish live from dead cells and by counting them in a Neubauer chamber. To perform a growth curve, parasites from three independent experiments were counted. Parasites were induced to encyst as described above. 2-BP or 2-FP were added with encysting media for 48 h to reach a final concentration of 10, 20 or 40 µM for 2-BP, and 100 µM for 2-FP. The inhibitors were diluted in DMSO as mentioned above. For immunofluorescence the parasites were subcultured onto 12 mm round glass coverslips (Glaswarenfabrik Karl Hecht, Sondhein, Germany) in 24-well culture plates for 1 h, fixed with 4% paraformaldehyde in PBS for 20 min at 4°C, washed twice in PBS and blocked with 10% normal goat serum (Invitrogen, Carlsbad, CA) in 0.1% Triton X-100 in PBS for 30 min at 37°C. The samples were then incubated with FITC labeled anti-CWP1 mAb (Waterborne Inc., New Orleans, LA) diluted 1∶250 in PBS containing 3% normal goat serum and 0.1% Triton X-100 for 1 h at 37°C or anti-CWP1 mAb and DAPI diluted in PBS (dilution 1∶500) (Sigma, St. Louis, MO). The coverslips were then mounted onto glass slides using FluorSave reagent (Calbiochem, La Jolla, CA). Fluorescence was visualized in a Zeiss Axiovert 200 microscope (Carl Zeiss, Jena, Germany). To quantify the percentage of encysting parasites, 55 cells from three separate experiments were counted and classified as encysting I, encysting II, or cyst according to the cell shape, membrane staining, and number and size of the encystation-specific vesicles. The average was taken in each of the three groups.

### Dataset construction, multiple sequence alignment, and phylogenetic analyses

A proteome database was constructed gathering complete proteomes for 25 Metazoa (*Amphimedon queenslandica* (aqu), *Anolis carolinensis* (aca), *Apis mellifera* (apm), *Bombyx mori* (bmo), *Caenorhabditis elegans* (cae), *Canis familiaris* (cfa), *Ciona intestinalis* (cin), *Danio rerio* (dre), *Daphnia pulex* (dpu), *Drosophila melanogaster* (dme), *Equus caballus* (eqc), *Felis catus* (fca), *Gallus gallus* (gga), *Gorilla gorilla* (ggo), *Homo sapiens* (hsa), *Ixodes scapularis* (ixs), *Mus musculus* (mmu), *Nematostella vectensis* (nve), *Ornithorhynchus anatinus (oan), Petromyzon marinus* (pma), *Pteropus vampyrus* (pva), *Rattus norvegicus* (rno), *Schistosoma mansoni* (sma), *Sus scrofa* (ssc) and *Xenopus tropicalis* (xtr)), 18 Fungi (*Aspergillus nidulans* (and), *Batrachochytrium dendrobatidis* (bde), *Botryotinia fuckeliana* (bfu), *Candida albicans* (clb), *Encephalitozoon cuniculi* (ecu), *Gibberella zeae* (gze), *Leptosphaeria maculans* (lem), *Nematocida sp* (nsp), *Neurospora crassa* (ncr), *Pichia pastoris* (ppa), *Puccinia graminis* (pug), *Saccharomyces cerevisiae* (sce), *Schizosaccharomyces pombe* (szp), *Sclerotinia sclerotiorum* (scl), *Tuber melanosporum* (tme), *Ustilago maydis* (uma), *Vittaforma corneae* (vco) and *Yarrowia lipolytica* (yli)), 12 Plants (*Arabidopsis thaliana* (ath), *Brachypodium distachyon* (bdi), *Glycine max* (gmx), *Medicago truncatula* (met), *Oryza sativa* (osa), *Physcomitrella patens* (php), *Populus trichocarpa* (pot), *Selaginella moellendorffii* (smo), *Solanum lycopersicum* (sly), *Solanum tuberosum* (stu), *Sorghum bicolor* (sbi) *and Vitis vinifera* (vvi)), 1 Brown alga (*Aureococcus anophagefferens* (aan)), 1 Red alga (*Cyanidioschyzon merolae* (cym)), 3 Green algae (*Ostreococcus taurii* (ota), *Chlamydomonas reinhardtii* (chr) and *Chlorella variabilis* (chv)), and 24 Protists (*Babesia bovis* (bbo), *Bigelowiella natans* (bna), *Chlamydomonas reinhardtii* (chr), *Chlorella sp* (chl), *Cryptosporidium parvum* (cpv), *Dictyostelium discoideum* (ddi), *Entamoeba histolytica* (ehi), *Giardia lamblia* (gla), *Guillardia theta* (gth), *Leishmania major* (lma), *Paramecium tetraurelia* (pat), *Perkinsus marinus* (pem), *Phaeodactylum tricornutum* (pht), *Phytophthora capsici* (pcs), *Phytophthora ramorum* (pra), *Plasmodium falciparum* (pfa), *Polysphondylium pallidum* (pop), *Tetrahymena thermophila* (tet), *Thalassiosira pseudonana* (thp), *Theileria parva* (thp), *Toxoplasma gondii* (tgo), *Trichomonas vaginalis* (tva), *Trypanosoma brucei* (trb) and *Trypanosoma cruzi* (tcz)) from Ensembl, the Joint Genome Institute (JGI) and the NCBI databanks. zf-DHHC HMMer profile was obtained from Pfam [46], and used to search the proteomes database [47]. Incomplete sequences or those that did not start with the M residue were deleted from the dataset. Also, 90% similar amino acid sequences were clustered using CD-HIT web server with default settings, to reduce the redundancy of the set [48]. The final dataset contained 1034 amino acid sequences. Multiple sequence alignment of DHHC-CRD amino acid sequences was carried out using PROMALS3D online server with default settings [49]. Following manual curation using GeneDoc software [50], sequences lacking conservation in the regions of interest (i.e., DPG, DHHC-CRD and TTxE) were removed. Block Mapping and Gathering with Entropy (BMGE) [51] was used to select columns suitable for phylogenetic inference with the following settings: m = BLOSUM30, g = 0.2, b = 4.

Phylogenetic analysis was performed by Maximum Likelihood (ML) using PhyML [52] with approximate likelihood-ratio test (aLRT), in combination with the LG+G amino acid replacement matrix, which was determined by ProtTest to be the model of protein evolution which best fit the data [53]. Phylogenetic trees were generated and edited with Itol [54].

### Semiquantitative Reverse Transcription Polymerase Chain Reaction (RT-PCR)

RNA from WB1267 trophozoites or 48 h encysting WB1267 was extracted and purified using TRIzol reagent (Invitrogen, Carlsbad, CA) and SV total RNA Isolation System (Promega, Madison, WI). Total RNA were reverse transcribed using Revertaid reverse transcriptase according to the manufacturer's specifications (Fermentas, Thermo Scientific, PA). DNA contamination was tested by performing PCR in a “-RT” control (a mock reverse transcription containing all the RT-PCR reagents, except the reverse transcriptase. For PCR, 30 cycles (30 s at 94°C, 30 s at 55°C and 1 min at 72°C) were used ending with a final extension of 10 min at 72°C. The expression of the *Giardia* glutamate dehydrogenase *(gdh)* gene was assayed for positive control. Aliquots (50 µl) of the RT-PCR reaction were size-separated on 1% agarose gel prestained with SYBR Safe (Invitrogen, Carlsbad, CA). Primers sequences used in RT-PCR are displayed in table S2. These assays were performed four times in duplicates.

### Relative quantitative Real Time-PCR (qRT-PCR)

RNA from WB1267 trophozoites, 48 h encysting WB1267 or *dhhc* transgenic 48 h encysting cells (GL50803_1908, GL50803_2116, GL50803_16928, GL50803_8711) was extracted and purified as described above. 2 µg of total RNA were reverse transcribed using Revertaid reverse transcriptase according to the manufacturer's specifications (Fermentas, Thermo Scientific, PA). DNA contamination was tested as described above. cDNA samples were stored at −80°C until use. Control samples were prepared as above using nuclease-free ddH2O in place of RNA. Primers for PCR were designed using Primer express 3.0 software (Applied Biosystems, Forster City, CA) and were synthesized by Invitrogen, Inc. (Carlsbad, CA). Amplification was performed in a final volume of 20 µl, containing 2 µl of each cDNA sample which were previously diluted 1∶1000 (for *dhhc* genes) or 1∶10000 (for *cwp* genes), and 10 µl of SYBR Green Master Mix (Applied Biosystems, Foster City, CA). qRT-PCR was performed in a StepOne thermal cycler (Applied Biosystems, Foster City, CA). The mRNA levels of the genes studied were normalized to the expression of the *Giardia* glutamate dehydrogenase (*gdh*) gene. The relative-quantitative RT-PCR conditions were: holding stage: 95°C for 10 min, cycling stage: 40 cycles at 95°C for 15 s, 60°C for 1 min and melt curve stage: 95°C for 15 s, 60°C for 1 min, and 95°C for 15 s. Expression data were determined by using the comparative ΔΔCt method [55]. Primer sequences used in qRT-PCR are displayed in table S3.

### Western blot analysis

For Western Blot assays, parasite lysates or purified palmitoylated proteins were incubated with 2× Laemmli buffer, boiled for 10 min, and separated in 10% Bis-Tris gels using a Mini Protean II electrophoresis unit (Bio-Rad). Samples were transferred to nitrocellulose membranes (GE Healthcare Biosciences, Pittsburgh, PA), blocked with 5% skimmed milk and 0.1% Tween 20 in PBS, and later incubated with anti-HA mAb or anti-V5 mAb (Sigma, St. Louis, MO; dilution 1∶1000 or 1∶50 respectively) diluted in the same buffer for 1 h. The membrane was then washed, incubated with IDRye 800CW conjugated goat anti-mouse Ab (LI-COR, Lincoln, NE; dilution 1∶10000) for 1 h, and analyzed on the Odyssey scanner (LI-COR, Lincoln, NE). For the analysis of VSPs expression, blockage was performed with 5% skimmed milk and 0.1% Tween 20 in TBS, and then incubated with 5C1 anti-VSP1267 mAb diluted in the same buffer for 1 h. After washing and incubation with an enzyme-conjugated secondary antibody, proteins were visualized with the SuperSignal West Pico Chemiluminescent Substrate (Pierce, Thermo Fisher Scientific Inc., Rockford, IL, USA) and autoradiography. Controls included the omission of the primary antibody, the use of an unrelated antibody, or assays using non-transfected cells.

### Immunofluorescence

For immunofluorescence assays (IFA), trophozoites or encysting cells cultured in growth medium or encysting medium, respectively, were harvested and washed two times with PBSm (1% growth medium in PBS, pH 7.4) and allowed to attach to multi-well slides in a humidified chamber at 37°C for 30 min. After fixation with 4% formaldehyde (Sigma, St. Louis, MO) in PBS for 40 min at room temperature, the cells were washed with PBS and blocked with 10% normal goat serum (Invitrogen, Carlsbad, CA) in 0.1% Triton X-100 in PBS for 30 min at 37°C. Cells were then incubated with the anti-HA mAb (Sigma, St. Louis, MO; dilution 1∶500) in PBS containing 3% normal goat serum and 0.1% Triton X-100 for 1 h at 37°C, followed by incubation with Alexa 546-conjugated goat anti-mouse (dilution 1∶500) secondary antibody at 37°C for 1 h. Encysting cells were also incubated with FITC-conjugated anti-CWP1 mAb (Waterborne Inc., New Orleans, LA; dilution 1∶250). Alternatively, cells were incubated with 9C3 anti-BiP mAb (marker for ER) [56] or 5D2 anti-AP2 mAb (marker for peripheral vacuoles) [57] in PBS containing 3% normal goat serum and 0.1% Triton X-100 for 1 h at 37°C, followed by incubation with Alexa 546-conjugated goat anti-mouse (dilution 1∶500) secondary antibody at 37°C for 1 h. Samples were then incubated with FITC-conjugated anti-HA mAb (Sigma, St. Louis, MO; dilution 1∶100). Preparations were stained with DAPI diluted in PBS (dilution 1∶500) (Sigma, St. Louis, MO). Finally, preparations were washed with PBS and mounted in Vectashield mounting medium (Vector Laboratories, Burlingame, CA). Fluorescence staining was visualized with a motorized FV1000 Olympus confocal microscope (Olympus UK Ltd, UK), using 63× or 100× oil immersion objectives (NA 1.32). The fluorochromes were excited using an argon laser at 488 nm and a helio-neon laser at 543 nm. Detector slits were configured to minimize any cross-talk between the channels. Differential interference contrast images were collected simultaneously with the fluorescence images, by the use of a transmitted light detector. Images were processed using Fiji software [58] and Adobe Photoshop 8.0 (Adobe Systems) software. The colocalization and deconvolution were also performed using Fiji.

### Flow cytometry analysis

For the analysis of the amount of cyst yield in dhhc transgenic trophozoites by flow cytometry, the parasites were induced to encyst for 48 h. Trophozoites, encysting cells, and cysts were collected from confluent cultures. Parasites were pelleted by centrifugation at 1455 g for 15 min at 4°C, resuspended in cool sterile ddH2O and placed at 4°C overnight. Mature water-resistant cysts were then processed following the protocol for immunofluorescence (see above) without permeabilization. Briefly, parasites were washed two times with PBSm (1% growth medium in PBS, pH 7.4). After blockade with 10% normal goat serum, the parasites were labeled with anti-CWP1 mAb (Waterborne Inc, New Orleans, LA; dilution 1∶250) diluted in PBSm for 1 hour at 4°C. Cells were then washed twice in PBS and fixed with 4% formaldehyde (Sigma, St. Louis, MO) in PBS for 40 min at room temperature. Unlabeled samples were used to determine background fluorescence, and subsequently, fluorescently labeled cysts were analyzed in triplicate on a FACSCanto II flow cytometer (Becton & Dickinson, New Jersey, NY). All samples were analyzed in parallel by IFA to assess encystation efficiency.

### Statistics

Results were analyzed for statistical significance (defined as p<0.05 and indicated by asterisks in figures) by performing unpaired, two-sided Student's t-test with GraphPad Prism 5 Data Analysis Software (GraphPad Software, Inc., La Jolla, CA). Mean and standard error of mean (SEM) values were calculated from at least three biologically and technically independent experiments.

## Results and Discussion

### Growing and encysting parasites displayed a different pattern of total palmitoylated proteins with HCNCp and VSPs being palmitoylated during growth and encystation

**Figure 1:**
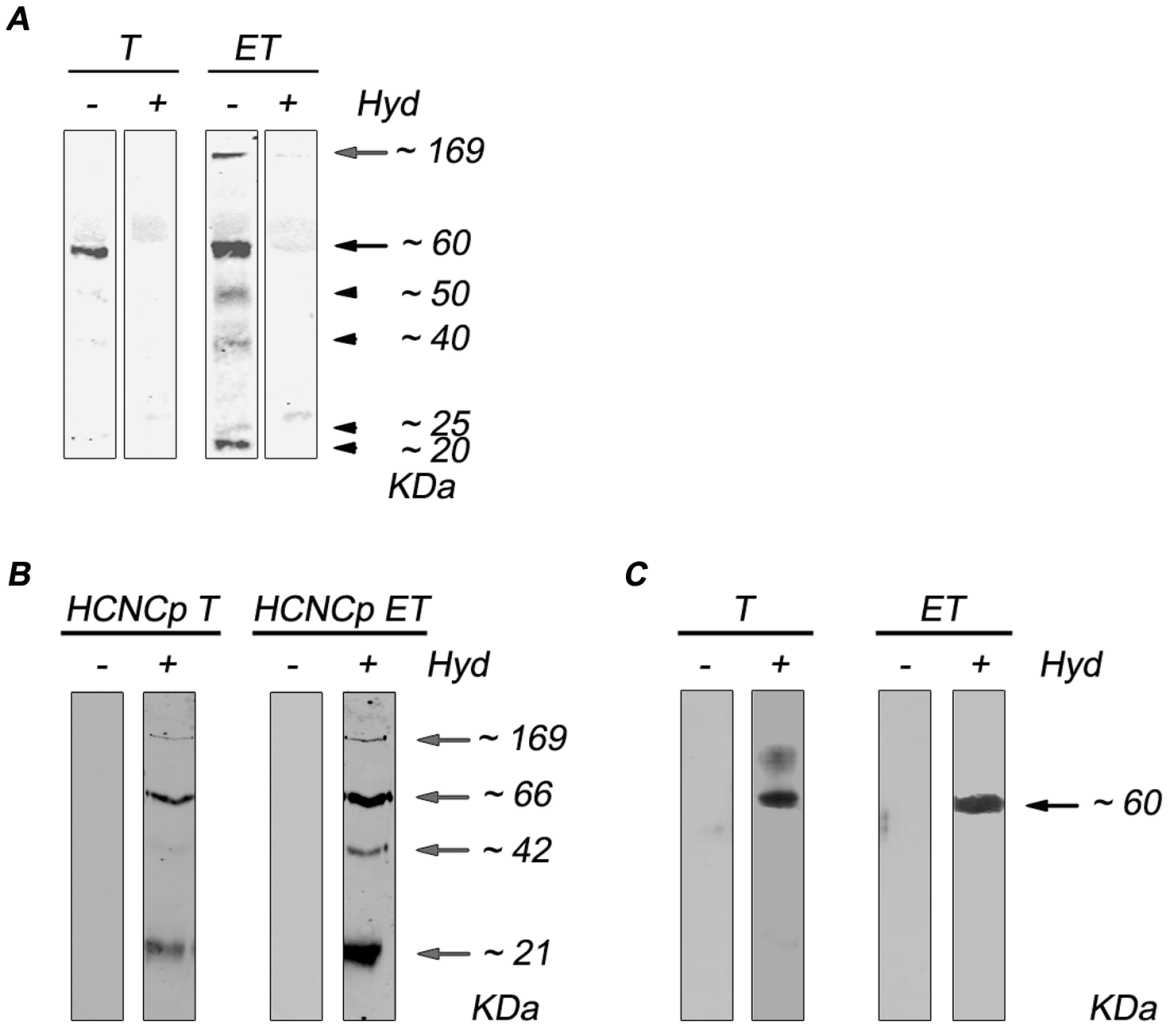
Analysis of S-palmitoylated proteins displays a different pattern in *Giardia* growing and encysting parasites. (A) *Giardia* trophozoites (T) or encysting trophozoites (ET) were labeled with [^3^H]-palmitic acid and loaded onto SDS-PAGE. The gel was treated with (hyd+) or without (hyd−) the thioester cleavage reagent hydroxylamine. Samples were then analyzed by autoradiography. (B) Western blotting performed on palmitoylated proteins purified by ABE from *hcncp-V5* transgenic trophozoites (HCNCp T) or *hcncp-V5* transgenic encysting trophozoites (HCNCp ET). (C) Western blotting performed on palmitoylated proteins purified by ABE from wild-type trophozoites (T) or encysting parasites (ET). The approximate sizes are indicated on the right in kDa.

It has been shown that protein palmitoylation actively participates in cell differentiation in a variety of cells [59], [60], [61]. The analysis of the expression of palmitoylated proteins, using metabolic labeling with [^3^H] palmitic acid, showed that encysting *Giardia* parasites displayed a different pattern of total protein palmitoylation than growing parasites (Figure 1A, T-ET/hyd−). The results showed a band of ∼60 kDa in trophozoites that may correspond to the expressed VSPs [31] (Figure 1A, T/hyd−). However, when *Giardia* encysting cells were analyzed, the assay displayed a larger amount of palmitoylated proteins, as can be judged by the larger number of bands displayed compared to trophozoites (Figure 1A, ET/hyd−). When we performed neutral treatment with hydroxylamine, almost complete removal of the attached palmitates was observed in both growing and encysting parasites (Figure 1A, T-ET/hyd+). This confirms that palmitate is attached through a labile thioester linkage (S-palmitoylation) in *Giardia*, as has been observed in other cell types including parasites [62], being most common among palmitoylated proteins [63]. Protein S-palmitoylation reversibility makes it a flexible, rapid and precise way of protein activity regulation [64] which may be crucial in the encystation process. The fact that the amount of total S-palmitoylated proteins was higher in encysting cells compared to trophozoites suggested that this PTM may play an important role during *Giardia* differentiation. This observation is in accordance with previous reports showing an important role of protein S-palmitoylation in controlling several crucial processes in parasites such as invasion or motility [44]. During *Giardia* encystation, the cyst wall proteins (CWPs) are sorted, concentrated within encystation-specific vesicles (ESVs), and exported to the nascent cyst wall [65], [66], [67]. Thus, the larger amount of palmitoylated proteins observed in encysting parasites (Figure 1A, ET/hyd−) may be explained by this additional requirement of protein sorting and export during this stage. In addition to the CWP1, 2 and 3, another type of cyst wall protein has been identified, a High Cysteine Non-variant Cyst protein (HCNCp) [68]. HCNCp belongs to a large group of cysteine-rich, non-VSPs, Type I integral membrane proteins (HCMp) [68]. The palmitoylation prediction algorithm CSS-Palm 3.0 [69] strongly predicts that HCNCp is palmitoylated at cysteines 1602 (CSS-Palm score 6.57, high stringency cut-off 0.31) and 1603 (CSS-Palm score 4.99, high stringency cut-off 0.31), which are located in the transmembrane region and in the cytosolic tail respectively (HMMTOP, (http://www.enzim.hu/hmmtop/) [70], [71]). In order to find out whether HCNCp is palmitoylated or not, we performed the following approach: first, we expressed full length HCNCp as a fusion protein containing a C-terminal V5-tag and a tubulin promoter [39]. The expression of the ∼169 kDa HCNCp protein was equally observed in *hcncp-V5* transgenic growing and encysting parasites, together with fragments of 21, 42 and 66 kDa already reported by Davids et al. [68] (Figure S1). Second, *hcncp-V5* transgenic trophozoites (HCNCp T) and encysting (HCNCp ET) parasites were subjected to acyl biotin exchange (ABE) as described in Methods. Parallel plus- and minus-hydroxilamine (hyd) samples were analyzed by Western blotting using an anti-V5 mAb (Figure 1B). Only the samples that were treated with hydroxylamine had free cysteine residues able to be detected by biotin/streptavidin (see Methods). When we assayed HCNCp T purified samples, we observed three bands (169, 66 and 21 kDa) and a weak band of 42 KDa (Figure 1B, HCNCp T/hyd+). Also, the four bands (169, 66, 42, and 21 kDa) were observed for HCNCp ET purified sample compared to the control (hyd−), showing that not only the full length but also the smaller epitope-tagged fragments of the HCNCp protein were palmitoylated in encysting parasites (Figure 1B, HCNCp ET/hyd+). The presence of these four bands may account, at least in part, for the bands shown in figure 1A (Figure 1A, ET/hyd−). Although we showed that the constitutively expressed HCNCp can be palmitoylated during growth and encystation, it was clearly reported that HCNCp is almost exclusively expressed during encystation when its expression was analyzed at the mRNA and protein (expression under its own promoter) levels [68]. Altogether, these results suggest that HCNCp is likely important during encystation, while the machinery necessary for its palmitoylation remains unaltered during growth and differentiation. Despite the need of additional assays to accurately identify additional palmitoylation substrates, it seems that this PTM is more frequently founded in encysting cells compared to trophozoites. In parallel to HCNCp T and HCNCp ET samples, we also performed ABE in wild-type trophozoites and encysting parasites and analyzed the purified samples by Western blotting using anti-VSP1267 mAb (Figure 1C). The results showed the specific protein band of VSP1267 (MW ∼60 KDa), in both growing and encysting parasites, suggesting that this PTM may be important for VSP function during the entire *Giardia* life cycle.

Further analysis using ABE or click chemistry [72] assays, together with different methods for Mass spectrometry-based proteomics, including Multidimensional protein identification technology [45], will expand our knowledge about other palmitoylated proteins in *Giardia*, defining the palmitoyl proteome of this parasite and shedding light on the role of this PTM in its life cycle.

### Inhibition of palmitoylation during *Giardia* encystation yielded a low number of cysts

**Figure 2:**
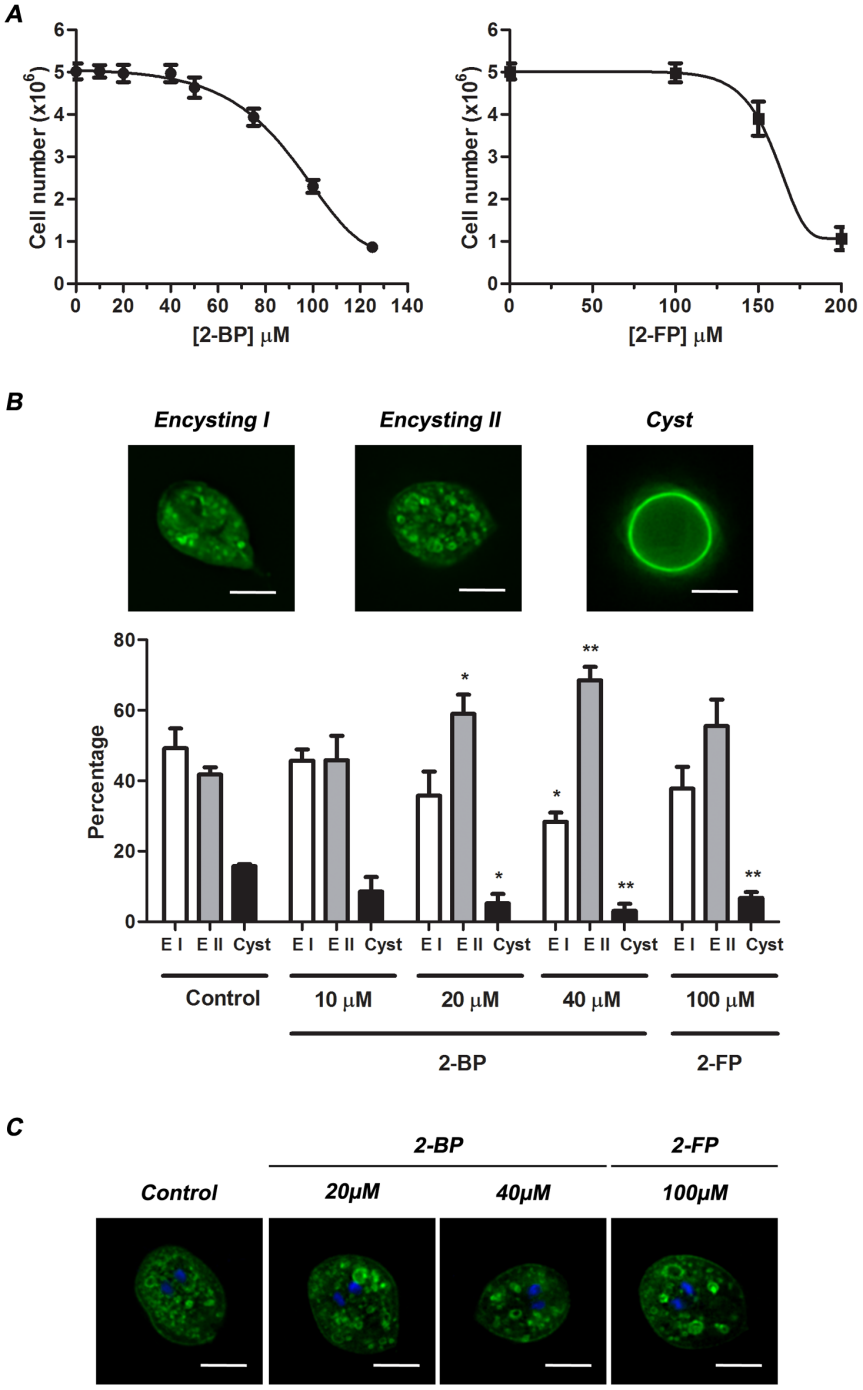
Inhibition of protein palmitoylation yields a low amount of *Giardia* cysts. (A) Growth curves displaying optimal concentrations of 2-BP (left panel) or 2-FP (right panel) that do not affect *Giardia* growth. *Giardia* trophozoites were cultured with different concentrations of 2-BP (10, 20, 40, 50, 75 or 100 µM), 2-FP (100, 150 or 200 µM), or DMSO (control) for 48 h. The parasites were then analyzed by staining them with Trypan blue to distinguish live from dead cells and by counting them in a Neubauer chamber. The graph displays the number (mean ± SEM) of parasites counted in three independent experiments. (B) Percentage of encysting parasites and cysts after inhibition of protein palmitoylation. *Giardia* trophozoites were induced to encyst and 2-BP (10, 20 or 40 µM), 2-FP (100 uM) or DMSO (Control) added to the encysting media. After 48 h, the encysting parasites were stained with anti-CWP1 mAb and analyzed by fluorescence microscopy. One representative cell of each encystation state (encysting I, encysting II, cyst) is shown in the upper panel. The graph in the lower panel represents the percentage (mean + SEM) of the cells counted in each state in three independent experiments. The asterisks indicate significant difference compared with the control (Student's t test: * p<0.05; **p<0.01; ***p<0.001). (C) Number of nuclei in encysting II parasites treated with palmitoylation inhibitors. Trophozoites were induced to encyst and 2-BP (20 or 40 µM), 2-FP (100 µM) or DMSO (Control) added to the encystation media as described above. After 48 h, the encysting parasites were stained with anti-CWP1 mAb and DAPI, and analyzed by fluorescence microscopy. One representative encysting II cell is shown. Scale bars = 5 µm.

The fact that *Giardia* encysting cells displayed a large amount of palmitoylated proteins prompted us to find out whether inhibition of protein palmitoylation would influence *Giardia* encystation. Several compounds have been reported to block protein palmitoylation [73]. The 2-bromopalmitate (2-BP) [74] and the 2-fluoropalmitate (2-FP) [73] inhibitors are non-metabolizable palmitate analogs that block palmitate incorporation into proteins using a still unclear mechanism. These two compounds have been widely used, act as broad inhibitors of palmitate incorporation and do not appear to selectively inhibit the palmitoylation of specific protein substrates. To test the effect of these inhibitors during encystation, *Giardia* wild-type trophozoites were induced to encyst together with the addition of either 2-BP or 2-FP. It has been reported that 2-BP is not well tolerated by *in vitro* cultured cells and causes cell death even after a brief exposure to 100 µM of 2-BP [75]. Thus, a growth curve was performed to determine the optimal concentrations that do not affect *Giardia* growth (10, 20 or 40 µM for 2-BP and 100 µM for 2-FP), observing that trophozoites died under concentrations higher than 50 µM of 2-BP or 150 µM of 2-FP (Figure 2A). After 48 h of encystation, treated or control parasites were harvested, permeabilized, stained with anti-CWP1 mAb and analyzed by fluorescence microscopy (Figure 2B). Wild-type encysting trophozoites were classified as encysting I (EI) (corresponding to 6 h of encystation [76]), encysting II (EII) (corresponding to 12 h of encystation [76]), and cysts (corresponding to 24–48 h of encystation [76]) (Figure 2B, upper panel), based on the following features: cell shape, membrane staining, and number and size of the ESVs. As shown in figure 2B (lower panel), there was a significant reduction in the amount of cysts when parasites were treated with 2-BP (20 µM or 40 µM) or 2-FP (100 µM).

The effect of 2-BP as a generic palmitoylation inhibitor has been reported in a wide variety of cells [77], [74], [78] including parasites like *Toxoplasma gondii* [62], although the concentrations used were much higher than the ones we used in this work. Interestingly, with 20 and 40 µM of 2-BP, there was an increase of the encysting II parasites compared to the control, reaching its highest levels when the concentration of 2-BP was 40 µM and resulting also in a diminution of encysting I cells (Figure 2B, lower panel). Thus, the decrease in the amount of cysts may be at the expense of the arrest of the cells at the encysting II stage of differentiation. In order to find out whether the treatment with palmitoylation inhibitors affect DNA replication, we analyzed the number of nuclei in the population of EII cells that were increased, observing no differences compared to the control (Figure 2C). Although a pleiotropic effect of 2-BP cannot be excluded, it is very likely that the observed decrease in cyst formation is associated with the inhibition of palmitoylation and the subsequent defect in ESVs docking and fusion, as was shown to be the case for other cells [79], [80].

Some results have suggested that palmitoylation in cells may occur nonenzymatically, i.e. spontaneous formation of thioester linkage in the presence of palmitoyl-CoA [81]. However, studies in yeast showed that DHHC protein family-mediated palmitoylation accounted for most of the palmitoylated proteins found in this organism [79]. Therefore, we decided to explore the *Giardia* proteome to study the presence of DHHC proteins in this parasite.

### Bioinformatics revealed the presence of nine DHHC proteins in the *Giardia* proteome

**Figure 3:**
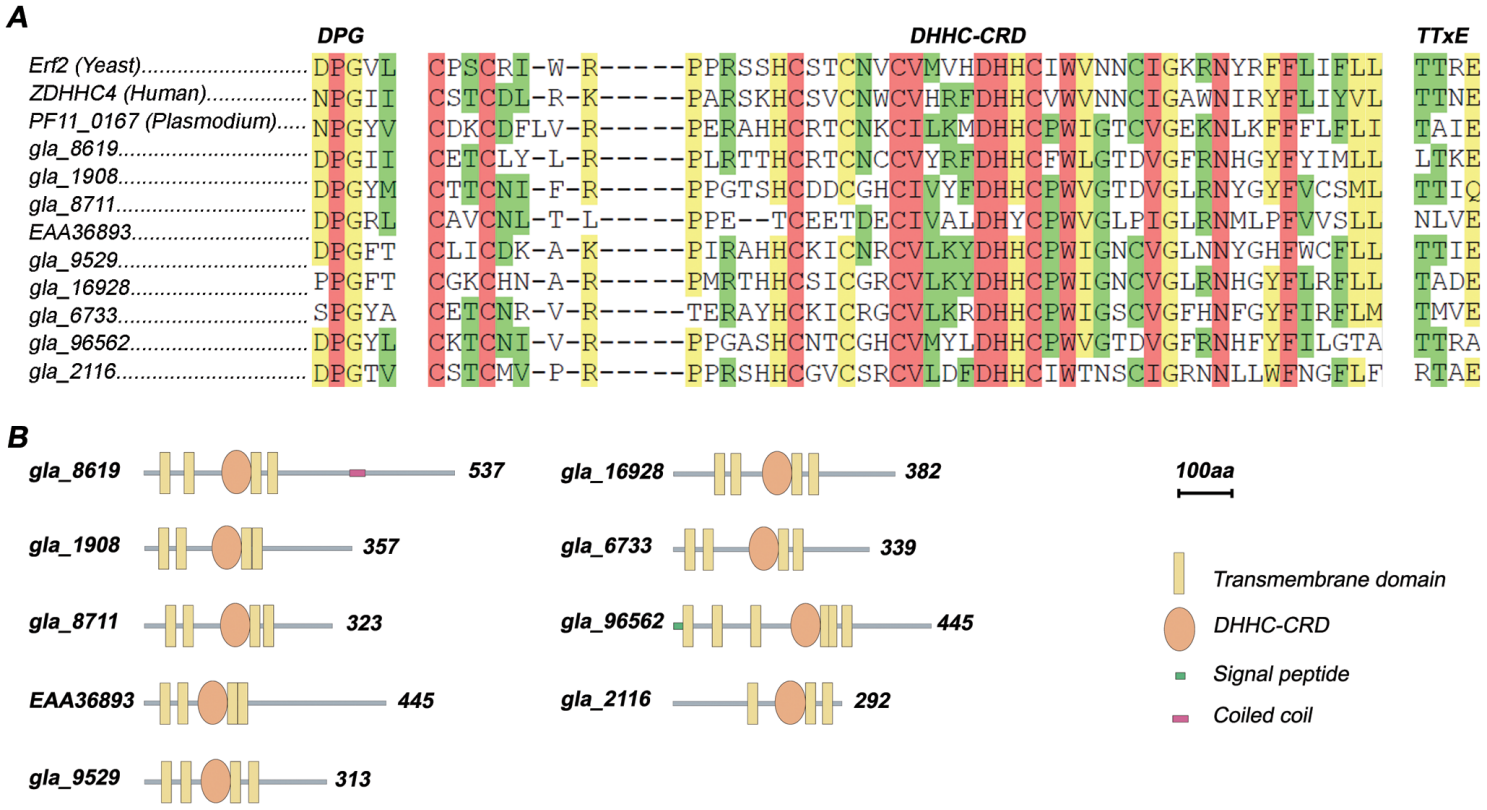
Sequence alignment and schematic drawing of *Giardia* DHHC proteins. (A) Multiple Sequence alignment of DHHC proteins shows conserved regions. The amino acid sequences of the total set of *Giardia* DHHC proteins, Erf2 (Yeast), ZDHHC4 (Human), and PF11_0167 (*Plasmodium falciparum*) were aligned using T-Coffee software [104]. The conserved DHHC-CRD domain and the DPG and TTxE motifs are indicated in bold. Positions exhibiting absolute identity are shown in pink, and high and lower amino acid similarities in green and yellow, respectively. (B) Schematic representation of the primary structure of *Giardia* DHHC proteins. The domains were searched using SMART (http://smart.embl-heidelberg.de) [105], [106]. Transmembrane domains were predicted using TMHMM (http://www.cbs.dtu.dk/services/TMHMM) [107] and TMPred (http://www.ch.embnet.org/software/TMPRED_form.html) with default settings. Signal peptides were predicted with signalP (http://www.cbs.dtu.dk/services/SignalP) [108].

PATs, the discovery of which has been crucial for the enzymology of palmitoylation, are a widespread evolutionary family of proteins [16], [82] ranging from eight in *Saccharomyces cerevisiae* [82], twelve in *Trypanosoma brucei* [27], eighteen in *Toxoplasma gondii* [25], twelve in *Plasmodium* [26], [25] to twenty-three members in humans [82]. To identify the complete set of *Giardia* putative PATs, we performed a HMMER search against the *Giardia* complete proteome using a DHHC PAT HMMer profile from Pfam (zf-DHHC). As shown in figure 3A, we found nine DHHC proteins in the *Giardia* proteome that displayed conserved sequences when compared to other organisms: i) the DHHC-CRD domain, ii) the two short motifs DPG (aspartate-proline-glycine) and iii) TTxE (threonine-threonine-any-glutamate) motif [20], [82]. One protein (gla_8711) contained a DHYC amino acid motif, instead of the canonical DHHC motif. However, this DHYC motif has been reported to be functional in the yeast PAT Akr1 [16].

We next analyzed the molecular identity of *Giardia* DHHC proteins with bioinformatics tools. In agreement with previous reports for other PATs [20], [18], [25], *Giardia* DHHC proteins were predicted to be polytopic membrane proteins, mainly harboring between three and six TMDs with the DHHC domain facing the cytosol (Figure 3B). There is a small group of DHHC proteins, including yeast DHHC protein Akr1, displaying the conserved 33 amino acid ankyrin repeats, which are frequently involved in protein-protein interactions [83]. By contrast, none of the *Giardia* DHHC proteins showed ankyrin repeats in their structure. Moreover, gla_8619 displayed a coiled coil structure and gla_96562 a signal peptide. As already described for other organisms [18], [25], *Giardia* DHHC proteins displayed a conserved structure, sharing domains and motifs that are present across all members of this enzyme family.

The names used in this paper, GiardiaDB, NCBI, and UniProt accession numbers for *Giardia* DHHC proteins are indicated in table 1.

**Table 1:** Collection of DHHC proteins in *Giardia lamblia*.

Name	GiardiaDB^1^ accession number	NCBI accession number	UniProt	Previously described (references)
gla_8619	GL50803_8619	XP_001704215	A8BY53	-
gla_1908	GL50803_1908	XP_001707652	A8BEE3	-
gla_8711	GL50803_8711	XP_001708375	A8BAE2	-
EAA36893	NFA^2^	EAA36893	A8BPQ2	(Touz et al., 2005)
gla_9529	GL50803_9529	XP_001709630	A8B4L4	(Touz et al., 2005)
gla_16928	GL50803_16928	XP_001706359	A8BKW0	(Touz et al., 2005)
gla_6733	GL50803_6733	XP_001707587	A8BE48	-
gla_96562	GL50803_96562	XP_001705995	A8BMZ6	-
gla_2116	GL50803_2116	XP_001704459	A8BW87	-

1 GiardiaDB version 3.1 [109].

2 NFA: not fully annotated.

The complete version of this protein is not annotated in GiardiaDB. It is only partially annotated as GL50803_42184 (Hypothetical protein sharing the last 252 aa with the *Giardia* PAT EAA36893 of 446 aa).

### Phylogenetic analysis of *Giardia* DHHC proteins

**Figure 4:**
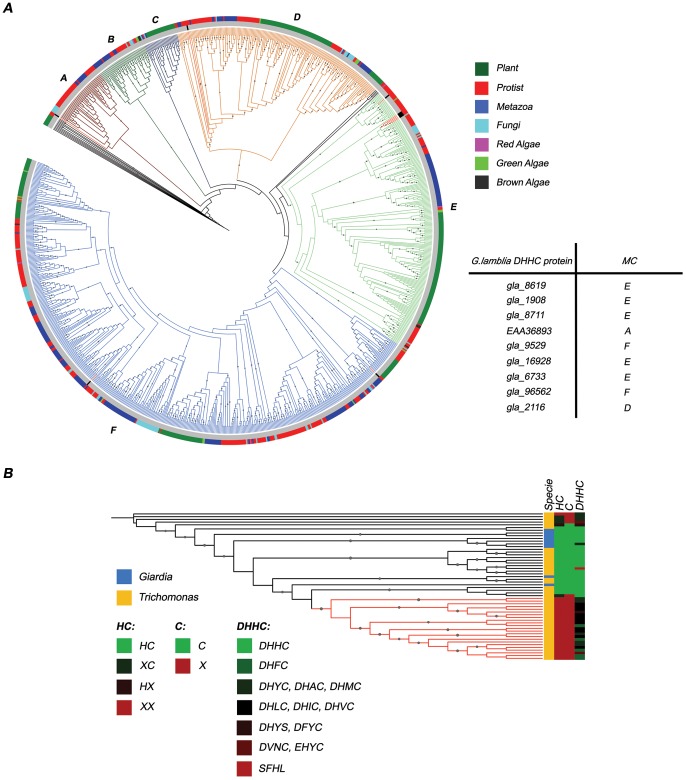
Phylogeny of DHHC proteins. (A) Phylogenetic relationships between DHHC proteins from *Giardia* and several other species. Phylogenetic tree of DHHC proteins inferred from ML analyses is depicted in the left panel. Symbols correspond to aLRT values >0.7. Sequence taxonomic identity is displayed with colors (outer circle around the tree), as shown in the upper right panel. MCs are labeled as A, B, C, D, E and F. *Giardia* DHHC proteins are colored in red and indicated in black in the inner circle around the tree. Each *Giardia* DHHC protein position in the tree (MC) is indicated in the table (lower right panel). (B) *Trichomonas* duplicated DHHC sequences accumulate mutations. *Giardia* DHHC proteins are indicated in light blue, and *Trichomonas* DHHC proteins in yellow. Variations in the HC, C, and DHHC portions of the DHHC-CRD domain were mapped in the tree using a green-to-black-to-red color code. Full conservation is depicted in light green, while lack of conservation is shown in red. A clade of highly mutated *Trichomonas* sequences is displayed in red.

In order to elucidate the phylogenetic relationship among the PATs and to infer the evolutionary history of *Giardia* DHHC proteins, we retrieved 1034 DHHC-CRD protein sequences from 84 completely sequenced eukaryotic genomes, including the *Giardia lamblia* genome (Assemblage A, isolate WB), by means of the DHHC PAT HMMer profile from Pfam (zf-DHHC). A Multiple Sequence Alignment was constructed with PROMALS3D [49], and Block Mapping and Gathering with Entropy (BMGE) [51] was used to select columns suitable for Maximum Likelihood (ML) phylogenetic inference. Maximum likelihood phylogenetic trees were calculated using PhyML [52], and Branch support was evaluated by approximate likelihood-ratio test (aLRT) [84]. The resultant phylogenetic tree can be divided in six monophyletic clades (MC), three of which together contain almost 90% of all sequences (MC D, E and F). Four MC have *Giardia* DHHC proteins: MC A and D contain one DHHC sequence each, while MC E and F contain five and two *Giardia* sequences respectively (Figure 4A and figures S2, S3, S4, S5). Without any further consideration than the topology of the tree and the early divergent phylogenetic status of *Giardia*, it can be argued that the Most Recent Common Ancestor of *Giardia* and the rest of the eukaryotic lineage (MRCA) had a minimum of four and a maximum of six groups of PATs. However, of the two *Giardia*-lacking MC one is almost entirely composed of Plant paralogues (MC C). Moreover, many MC contain subclades composed mostly or even only by Plant paralogues, suggesting that gene duplication have largely taken place in this group. All these can be seen as an indication of functional diversification among Plants, which also constitutes a plausible evolutionary mechanism for the origin of the MC C.

If we hypothesize that all DHHC sequences evolve from 4 PATs groups in the MRCA, we should be able to explain, in a parsimonious way, the MC lacking *Giardia* sequences as examples of evolutionary innovation. As we mentioned before, this is suitable in the case of the MC C, but not for the MC B (the other *Giardia* sequences-lacking MC). This is because MC B is composed of sequences from a greater variety of organisms compared to MC C, making the possibility of a common functional diversification very unlikely. Nevertheless, it is possible for the MC B to be the result of reductive evolution, meaning that *Giardia* lost sequences during its adaptation to a parasitic lifestyle, since the more stable environment provided by the host can cause relaxation or loss of selective constraints.

We tested gene loss across DHHC-CRD protein family by examining the heavily duplicated genomes of *Trichomonas vaginalis*, given that duplicated genes are most likely to be released from functional constraints (Figure 4B). For this, we retrieved all DHHC sequences from *Trichomonas* (http://trichdb.org/trichdb/) using the same pipeline described above, except that this time no sequences were excluded from the posterior analysis. Variations in the HC, C and DHHC portions of the DHHC-CRD domain were extracted from the MSA, and mapped onto a phylogenetic tree. Contrary to what is found in Plants, there is a substantial presence of poorly conserved sequences among *Trichomonas* genome that cluster together in the tree. Moreover, we found a strong correlation between the degree of conservation in the HC, C and DHHC portions of the DHHC-CRD domain within each sequence.

Altogether, our findings suggest that the MRCA had five groups of DHHC sequences from which the other sequences eventually evolved by functional diversification, and that *Giardia* lost at least one representative sequence presumably during its adaptation to a parasitic lifestyle.

**Figure 5:**
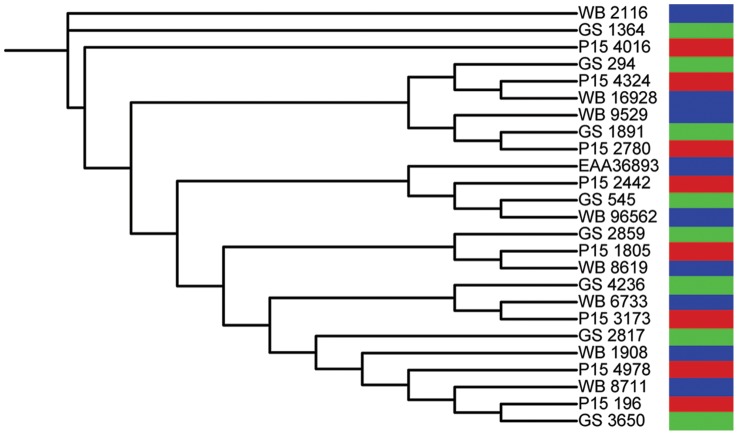
Orthology relationships between *Giardia* isolates WB, GS and P15 (Assemblages A, B and E, respectively). Phylogenetic tree of *Giardia* DHHC sequences from the three isolates inferred from ML analyses is depicted. Each isolate is indicated with a different color.

We also determined the orthology relationships between sequences from different assemblages. For this, we retrieved DHHC sequences from *Giardia* isolates WB, GS and P15 (Assemblages A, B and E, respectively; http://giardiadb.org/giardiadb/), following the pipeline described above. As expected, every DHHC sequence in the isolate WB has a highly similar ortholog in the other isolates, which cluster together in the tree (Figure 5). Only one WB sequence, EAA36893, escapes this pattern, but this probably constitutes a case of defective annotation in isolates GS and P15.

### DHHC proteins were expressed in trophozoites and encysting cells

**Figure 6:**
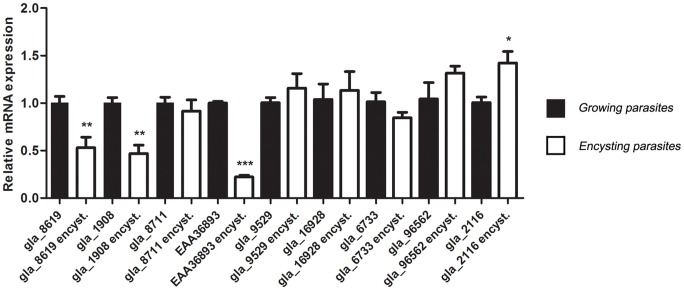
Differential expression of *Giardia dhhc* genes in growing and encysting parasites. Expression of gla_8619, gla_1908, gla_8711, EAA36893, gla_9529, gla_16928, gla_6733, gla_96562, gla_2116 transcripts from 48 h encysting parasites (white bars) relative to the expression in growing parasites (black bars). The data are the means and SEM of three separate experiments, and each experiment was carried out in triplicate. The qRT-PCR analysis of *dhhc* genes was performed as described in Methods. The asterisks indicate that there was significant difference compared with growing parasites (Student's t test: * p<0.05; **p<0.01; ***p<0.001).

Semi-quantitative RT-PCR indicated that all the *dhhc* genes were expressed in trophozoites and in encysting parasites (Figure S6). This prompted us to explore further the expression levels of these genes in growing and encysting parasites by performing qRT-PCR analysis of mRNA expression from these cells. As shown in figure 6, many of the *dhhc* transcripts were present at relatively constant levels, but gla_8619, gla_1908, and EAA36893 were downregulated in encysting parasites while gla_2116 was upregulated in 48 h encysting cells. Considering that *Giardia* contains minimal systems, either as a result of reductive processes associated with a parasitic lifestyle, as a reflection of basic evolutionary characteristics, or both [85], [86], the fact that the nine *dhhc* genes found by bioinformatics were expressed in vegetative and encysting parasites suggests that protein palmitoylation and the PATs themselves may be playing a key role during the entire life cycle of this parasite.

We next sought to characterize four of the nine DHHC proteins that are expressed in *Giardia* based on their expression profile. We chose two that are expressed at similar levels in growing and encysting parasites (gla_8711 and gla_16928), one that is downregulated during encystation (gla_1908), and one that is upregulated in encysting parasites (gla_2116).

### DHHC proteins gla_1908, gla_2116, gla_16928, gla_8711 displayed a different intracellular localization

**Figure 7:**
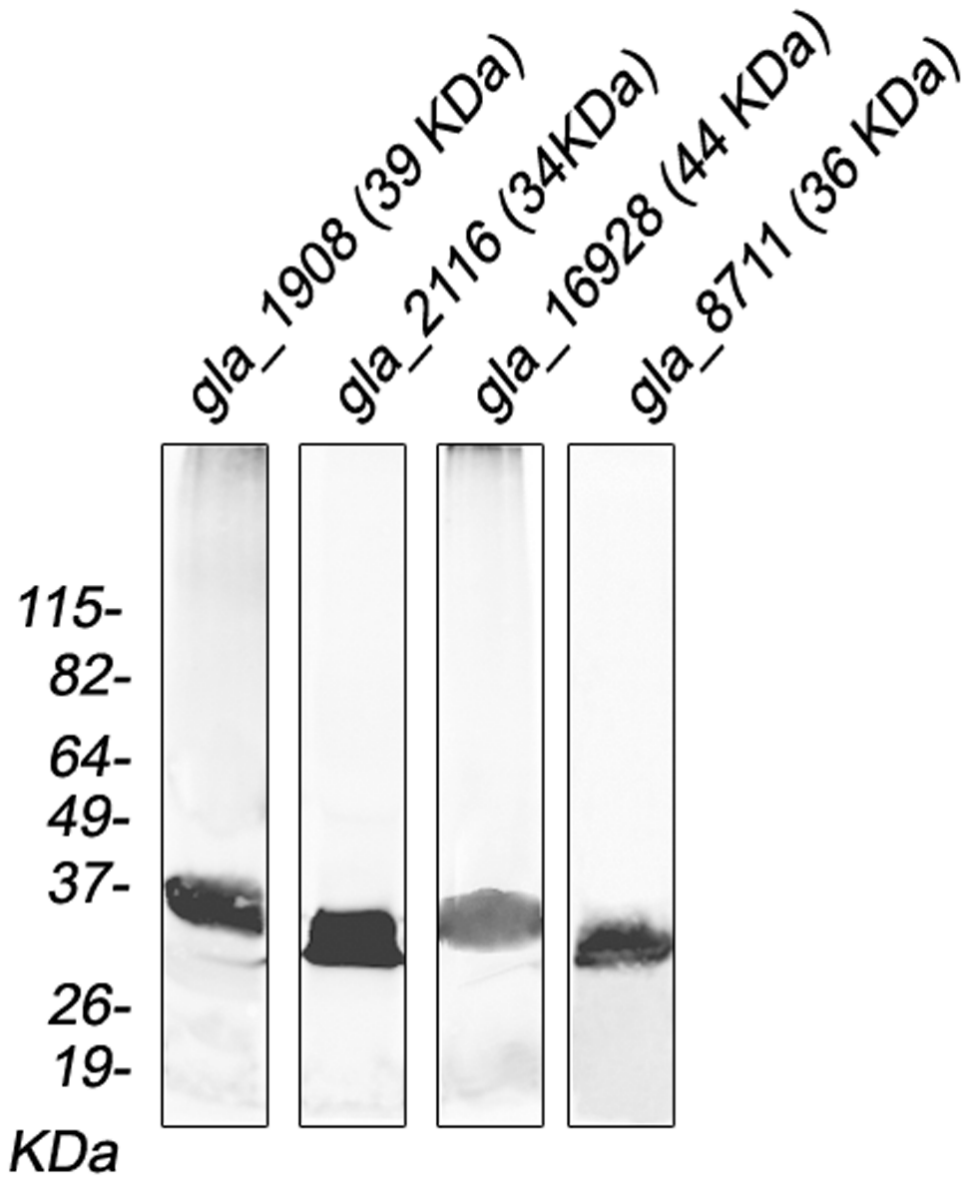
Expression of DHHC-HA proteins in *Giardia* trophozoites. Western blotting performed on total protein extracts from *dhhc-ha* transgenic trophozoites. Expected sizes are indicated in brackets. Relative molecular weights of protein standards (kDa) are indicated on the left.

**Figure 8:**
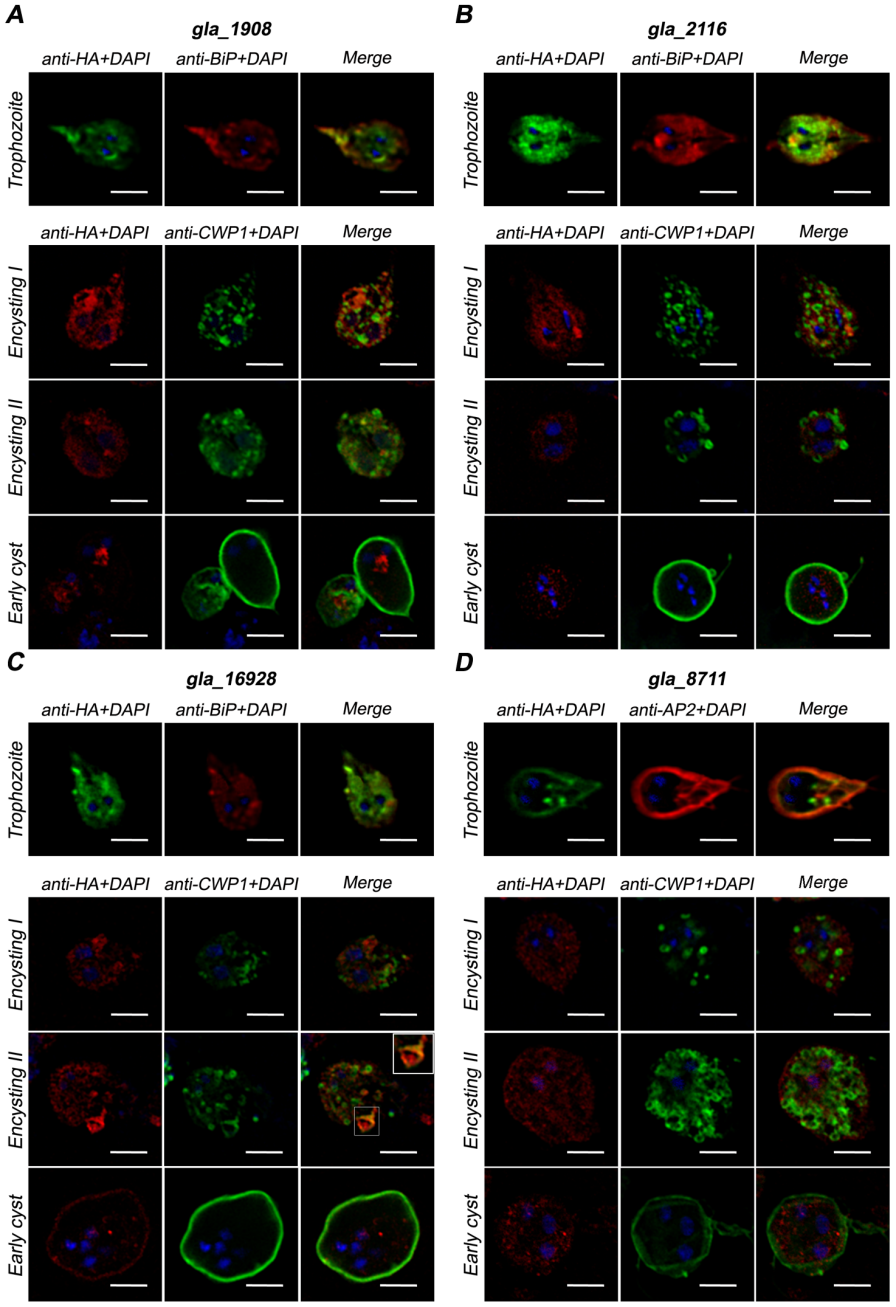
Localization of DHHC-HA proteins in trophozoites and effect of DHHC-HA overexpression in encystation. Subcellular localization of gla_1908-HA (A), gla_2116-HA (B), gla_16928-HA (C), or gla_8711-HA (D) in trophozoites or encysting parasites. For trophozoites, gla_1908-HA, gla_2116-HA or gla_16928-HA were stained with anti-BiP (ER) mAb, anti-HA mAb and DAPI; gla_8711-HA was stained with anti-AP2 (PVs) mAb, anti-HA mAb and DAPI. For encysting parasites, after 48 h of encystation *dhhc-ha* transgenic parasites were stained with anti-HA mAb, anti-CWP1 mAb and DAPI. The cells were analyzed by fluorescence microscopy. One representative cell from each stage is shown. Yellow areas in trophozoites indicate co-localization between DHHC-HA and ER (gla_1908-HA, gla_2116-HA or gla_16928-HA), or between DHHC-HA and PVs (gla_8711-HA). Yellow areas in encysting parasites indicate co-localization between DHHC-HA and CWP1. The inset in C (*gla_16928* transgenic encysting II parasites) corresponds to the zoomed area indicated by the lined box. Scale bars = 5 µm.

To further analyze these DHHC proteins, we expressed full-length gla_1908, gla_2116, gla_16928 and gla_8711 as fusion DHHC proteins containing C-terminal HA-tag [39] and evaluated their protein expression profiles by Western blotting using an anti-HA mAb (Figure 7). Analysis by semi-quantitative RT-PCR indicated that the overexpression of these fusion proteins was 2 to 3-times higher in transgenic cells, as reported for protein expression using a similar vector [9]. Immunofluorescence assays showed that HA-tagged gla_1908, gla_2116, and gla_16928 partially co-localized with BiP in the endoplasmic reticulum (ER) or around the nuclei of transgenic trophozoites (Figure 8, trophozoite). Our results confirmed the localization of gla_16928 already shown by Touz et al. [31]. Analysis of intracellular localization of yeast and mammalian DHHC proteins revealed that the majority of these localize to the ER and Golgi [20], [87]. However, there are a few exceptions, including human DHHC5 protein [87] and *Giardia* DHHC protein (EAA36893) [31], which localize to the plasma membrane. Also, we found that gla_8711 partially co-localized with the adaptor protein AP-2 [57] at the lysosomal-like peripheral vacuoles (PVs) as well as in plasma membrane and flagella (Figure 8, trophozoite). Ongoing experiments intended to knock-down this protein may reveal its importance during the *Giardia* life cycle.

### The overexpression of the DHHC proteins disclosed a differential involvement during encystation

The hallmark of encystation in *Giardia* is the synthesis of CWP1, CWP2, and CWP3 [88]. These proteins are expressed and concentrated within the ESVs before they are targeted to the cyst wall [89], [6], [90]. To address the influence of the overexpression of these HA-tagged DHHC proteins during encystation, *dhhc-ha* transgenic trophozoites were induced to encyst *in vitro*. The localization of DHHC-HA proteins as well as CWP1 expression, intracellular localization, and vesicle formation were addressed by IFA. To examine in detail the results obtained, we decided to analyze each *dhhc-ha* transgenic cell following the protocol described above, in which the cells were classified as encysting I, encysting II, and early cyst. We observed that *gla_1908* (Figure 8A), *gla_2116* (Figure 8B), and *gla_8711* (Figure 8D) transgenic parasites displayed normal encystation. It was noteworthy that *gla_16928* (Figure 8C) had enlarged ESVs, with co-localization between gla_16928-HA and CWP1 observed in those vesicles (Figure 8C, inset). Additionally, it was noted that *gla_16928* early cysts had a larger size and an abnormal shape compared with wild-type cells (not shown) and other transgenic early cysts.

**Figure 9:**
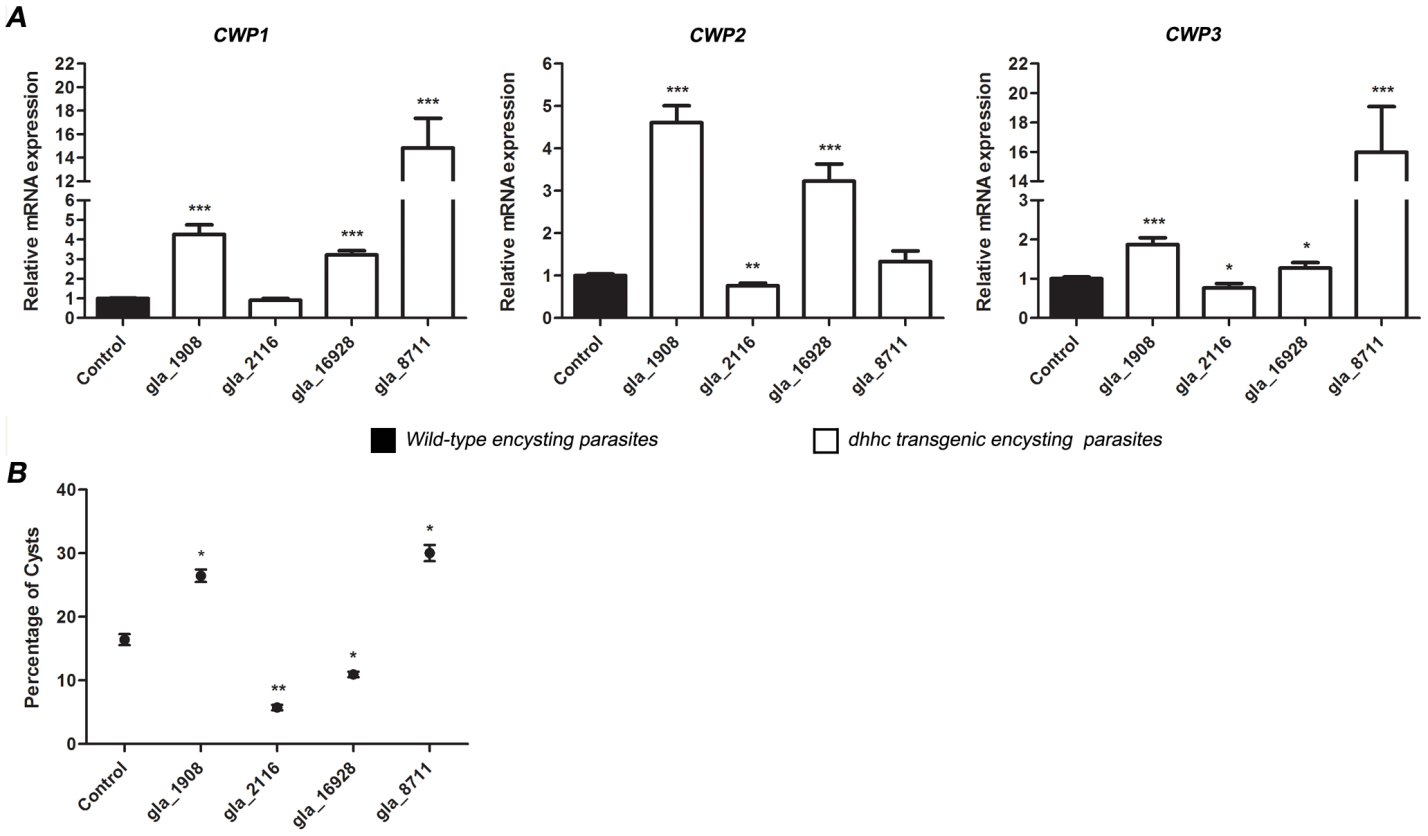
The expression of cyst wall protein transcripts and the amount of cysts are different among *dhhc* transgenic encysting parasites. (A) qRT-PCR analysis of *cwp1, cwp2*, and *cwp3* transcripts expression in *dhhc* transgenic parasites after 48 h of encystation (white bars), relative to the expression in wild-type encysting cells (control) (black bars). The data are the means and SEM of three separate experiments, and each experiment was carried out in triplicate. (B) Percentage of water-resistant cysts in *dhhc* transgenic parasites determined by flow cytometry after 48 h of encystation. The results are presented as the percentage (mean ± SEM) of cysts in three independent experiments. The asterisks indicate that there was significant difference compared with the control (Student's t test: * p<0.05; **p<0.01; ***p<0.001).

When CWP expression was analyzed in *dhhc* transgenic parasites by qRT-PCR, we observed that, except for *gla_2116* transgenic cells, which displayed similar levels or even moderate decrease in the mRNA expression of CWPs compared to the control, the other *dhhc-ha* transgenic parasites showed increased expression of CWP1, CWP2, and CWP3 (Figure 9A). Several transcription factors have been described as involved in the regulation of *cwp* gene transcription [91], [92], [93], [94], [95], [96], [97]. However, the mechanisms underlying transcription control in this parasite have not been completely elucidated. It has always been assumed that the mobilization mechanism for transcription factors in many organisms is based on proteolytic processing [98], [99], [100], [101]. Nevertheless, there is a group of lipid-modified transcription factors whose mobilization mechanism to the nucleus is not based on proteolytic processing but on reversible palmitoylation [102]. If that were the case for the transcription factors involved in *Giardia* encystation, DHHC proteins would be palmitoylating different transcriptions factors that, in turn, may regulate CWP expression. It would be interesting to explore the molecular architecture of *Giardia* transcription factors to find out whether palmitoylation is involved in regulating their shuttling between the cytoplasm and the nuclei.

Analyzing the amount of water-resistant cysts, we observed that *gla_1908* and *gla_8711* transgenic cells yielded a significantly higher amount of cysts than the control (Figure 9B). In contrast, *gla_2116* transgenic cells, while displaying an apparently normal encystation process (Figure 8B) and CWP expression (Figure 9A), produced a reduced number of mature cysts (Figure 9B). A likely explanation is that *gla_2116* may be involved in the palmitoylation of a protein in charge of turning encystation-specific genes off and ending the encystation process. In the case of *gla_16928* transgenic parasites, these cells produced a low percentage of cysts (Figure 9B) although the CWP expression was increased (Figure 9A). These findings, in addition to the large ESVs seen in figure 8C (encysting II) and the large size of early cysts (Figure 8C, early cyst), may be explained by a high rate of synthesis of CWPs in *gla_16928* transgenic parasites, which may exceed the mechanisms of vesicle discharge regulation, leading to the formation of immature non-water-resistant cysts. Further experiments using knock-down strategies are needed to completely address the role of each DHHC protein in the encystation process. Table 2 summarizes the main features of the *Giardia* DHHC proteins analyzed in this work.

**Table 2:** Main features of the *Giardia* DHHC proteins analyzed in this paper.

*dhhc* transgenic *Giardia* parasites	Gene expression	Subcellular localization	Development of encystation process observed by IFA	Expression of CWPs	Amount of mature water-resistant cysts produced
***gla_1908***	Reduced during encystation	ER and NE^1^	Normal	High	Large
***gla_2116***	Increased during encystation	ER and NE	Normal	Similar to wild type or even lower	Low
***gla_16928***	No significant difference	ER and NE	Large ESVs; large early cysts	High	Low
***gla_8711***	No significant difference	PM^2^	Normal	High	Large

1 NE: nuclear envelope.

2 PM: plasma membrane.

**Figure 10:**
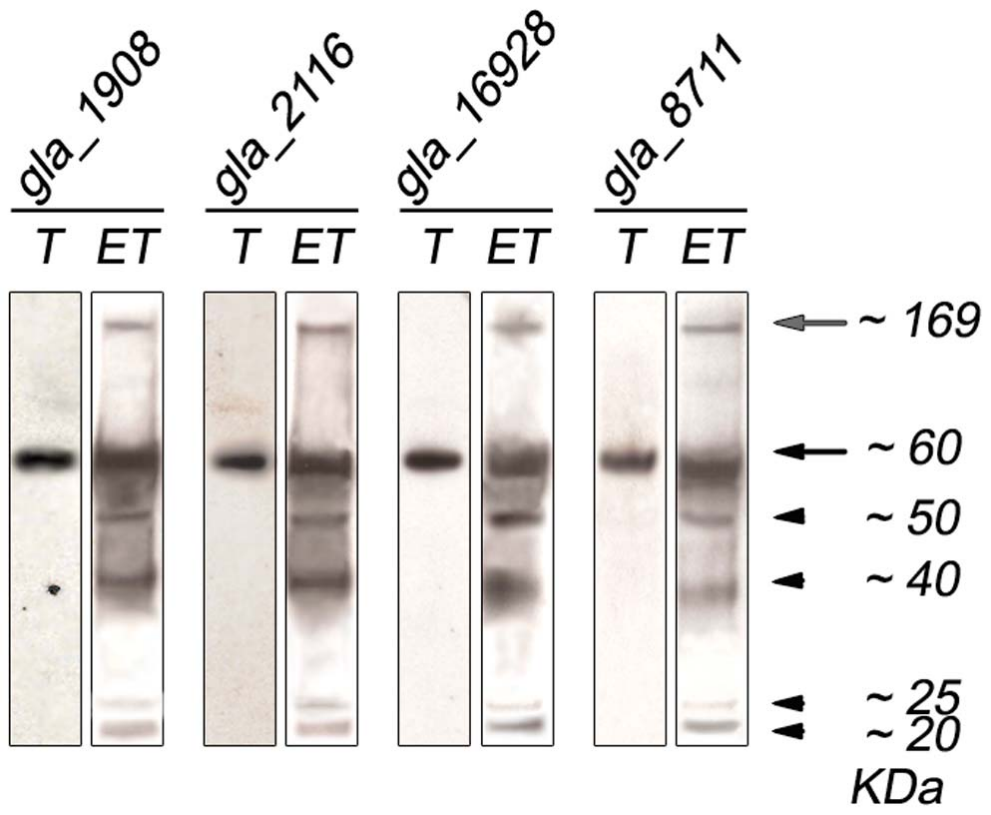
Analysis of palmitoylated proteins in *dhhc* transgenic growing and encysting parasites displays a similar pattern to wild type parasites. *Giardia* trophozoites (T) or encysting trophozoites (ET) were labeled with [^3^H]-palmitic acid and loaded onto SDS-PAGE. Samples were then analyzed by autoradiography. The approximate sizes are indicated on the right in kDa.

The different localization of DHHC-HA proteins in trophozoites and the differential effect of DHHC overexpression in encystation prompted us to evaluate the palmitoylation pattern in the *dhhc* transgenic parasites (Figure 10). *gla_1908, gla_2116, gla_16928*, and *gla_8711* transgenic trophozoites or encysting parasites displayed a similar global protein palmitoylation pattern compared to wild type (Figure 1A). Mass spectrometry-based proteomics analyses will be necessary to accurately identify any differences in the palmitoylation substrates among the *dhhc* transgenic parasites.

### Conclusion

This work presents a detailed analysis of *Giardia lamblia* DHHC protein structure and phylogeny and reveals a possible role of palmitoylation in *Giardia* encystation. Our data, suggesting the presence of DHHC proteins in growing and encysting parasites, reinforced the idea that this PTM has conserved and important functions in cell-signaling, protein-sorting and protein-export throughout evolution. Without being able to assign a specific substrate candidate to each *Giardia* DHHC proteins, we showed that overexpression of these enzymes had consequences on CWP expression and on the amount of cysts produced. Proteomic analysis of *Giardia* palmitoyl proteome would be a great contribution to elucidating the mechanisms by which palmitoylation participates in encystation biology. Finally, the suggested role of palmitoylation in *Giardia* encystation, a key event that enables the parasite to survive in the environment, infect a new host and evade the immune response [1], [103], could open new ways to intervene in the process of *Giardia* infection.

## Supporting Information

Figure S1 **Expression of HCNCp-V5 in** ***Giardia*** **growing and encysting parasites.** Western blotting performed on total protein extracts from *hcncp-V5* transgenic trophozoites (T) or *hcncp-V5* transgenic encysting trophozoites (ET). Expected size is indicated in brackets. Relative molecular weights of protein standards (kDa) are indicated on the left.
(TIF)

Figure S2 **The zoomed subclade containing gla_8619, gla_6733, gla_1908, and gla_8711 (A) or EAA36893 (B) from the phylogenetic tree presented in** **figure 4** **.** Sequence taxonomic identity is displayed with colors as described in figure 4.
(TIF)

Figure S3 **The zoomed subclade containing gla_9529 from the phylogenetic tree presented in** **figure 4** **.** Sequence taxonomic identity is displayed with colors as described in figure 4.
(TIF)

Figure S4 **The zoomed subclade containing gla_16928 (A) or gla_96562 (B) from the phylogenetic tree presented in** **figure 4** **.** Sequence taxonomic identity is displayed with colors as described in figure 4.
(TIF)

Figure S5 **The zoomed subclade containing gla_2116 from the phylogenetic tree presented in** **figure 4** **.** Sequence taxonomic identity is displayed with colors as described in figure 4.
(TIF)

Figure S6 **Differential expressions of** ***Giardia dhhc*** **genes in trophozoites and encysting parasites by semiquantitative RT-PCR.** Expression of gla_8619, gla_1908, gla_8711, EAA36893, gla_9529, gla_16928, gla_6733, gla_96562, gla_2116 transcripts from growing parasites (upper panel) and 48 h encysting parasites (lower panel). Expression of glutamate dehydrogenase *(gdh)* mRNA fragment was tested as positive control. Expected sizes are indicated in brackets. Relative molecular weights of standards (bp) are indicated on the left.
(TIF)

Table S1 **Oligonucleotide primers used for** ***Giardia*** **DHHC cloning.**
(DOCX)

Table S2 **Oligonucleotide primers used for semiquantitative RT-PCR.**
(DOCX)

Table S3 **Oligonucleotide primers used for qRT-PCR.**
(DOCX)
