# Correction: Identification of *Giardia lamblia* DHHC Proteins and the Role of Protein S-palmitoylation in the Encystation Process

**DOI:** 10.1371/journal.pntd.0003157

**Published:** 2014-08-14

**Authors:** The PLOS Neglected Tropical Diseases Staff

In Figure 3, Part A there is a sequence missing. The authors have provided a corrected version here.

**Figure 3:**
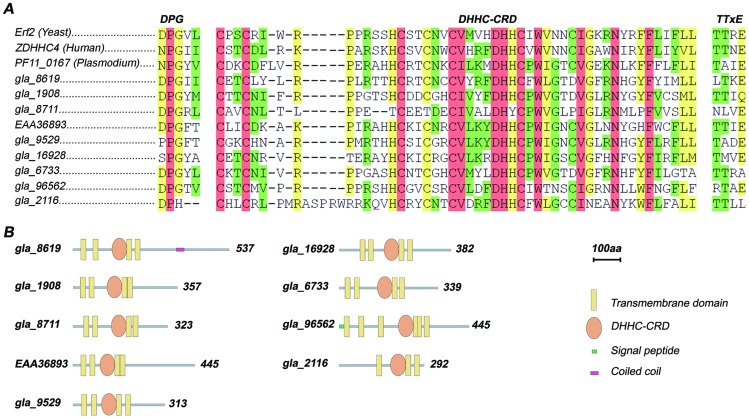
Sequence alignment and schematic drawing of *Giardia* DHHC proteins. (A) Multiple Sequence alignment of DHHC proteins shows conserved regions. The amino acid sequences of the total set of *Giardia* DHHC proteins, Erf2 (Yeast), ZDHHC4 (Human), and PF11_0167 (*Plasmodium falciparum*) were aligned using T-Coffee software [104]. The conserved DHHC-CRD domain and the DPG and TTxE motifs are indicated in bold. Positions exhibiting absolute identity are shown in pink, and high and lower amino acid similarities in green and yellow, respectively. (B) Schematic representation of the primary structure of Giardia DHHC proteins. The domains were searched using SMART (http://smart.embl-heidelberg.de) [105], [106]. Transmembrane domains were predicted using TMHMM (http://www.cbs.dtu.dk/services/TMHMM) [107] and TMPred (http://www.ch.embnet.org/software/TMPRED_form.html) with default settings. Signal peptides were predicted with signalP (http://www.cbs.dtu.dk/services/SignalP) [108].
