# Supplementary figures and images for: Identification of Giardia lamblia DHHC Proteins and the Role of Protein S-palmitoylation in the Encystation Process
Source: PLoS Negl Trop Dis. 2014 Jul 24;8(7):e2997. doi: 10.1371/journal.pntd.0002997 (PMC4109852; doi:10.1371/journal.pntd.0002997)

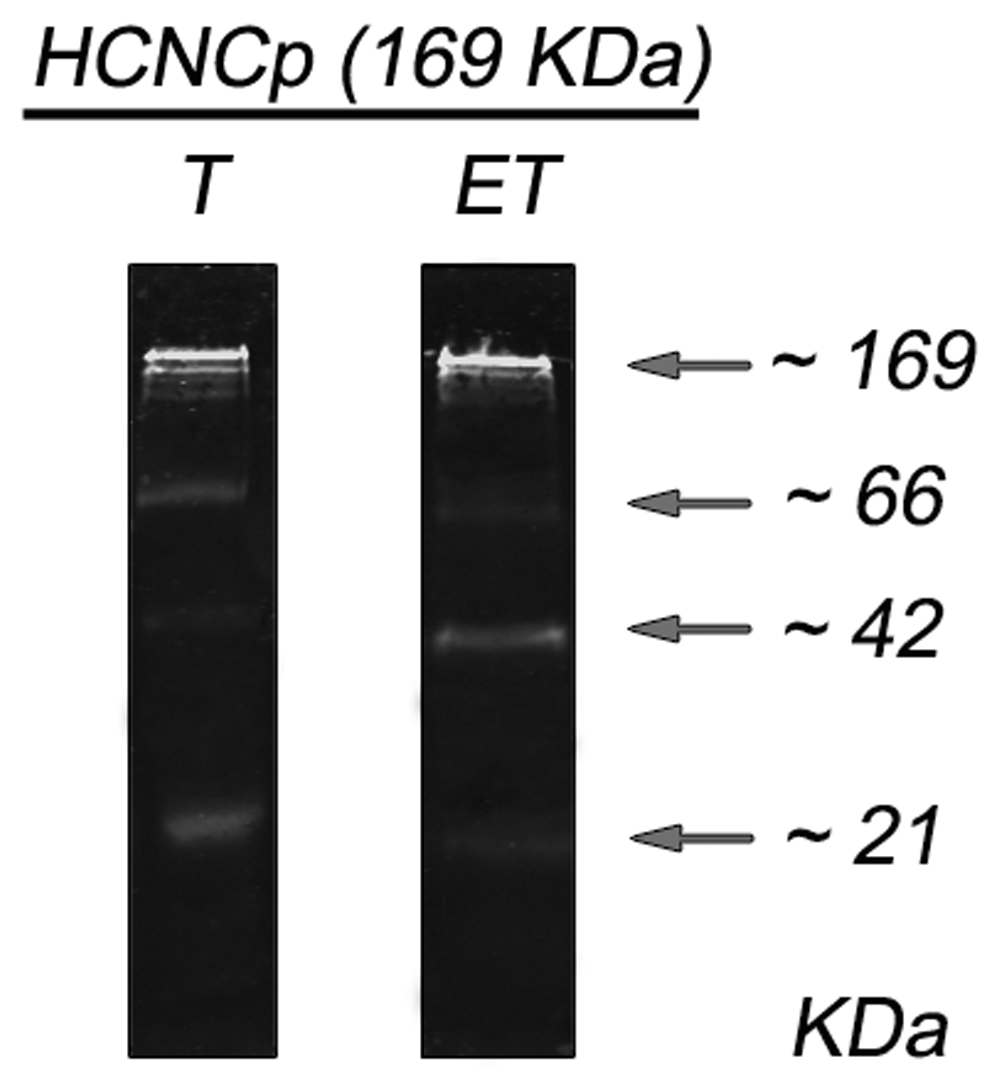

Supplement: Figure S1 — Expression of HCNCp-V5 in Giardia growing and encysting parasites. Western blotting performed on total protein extracts from hcncp-V5 transgenic trophozoites (T) or hcncp-V5 transgenic encysting trophozoites (ET). Expected size is indicated in brackets. Relative molecular weights of protein standards (kDa) are indicated on the left. (TIF) [file pntd.0002997.s001.tif]

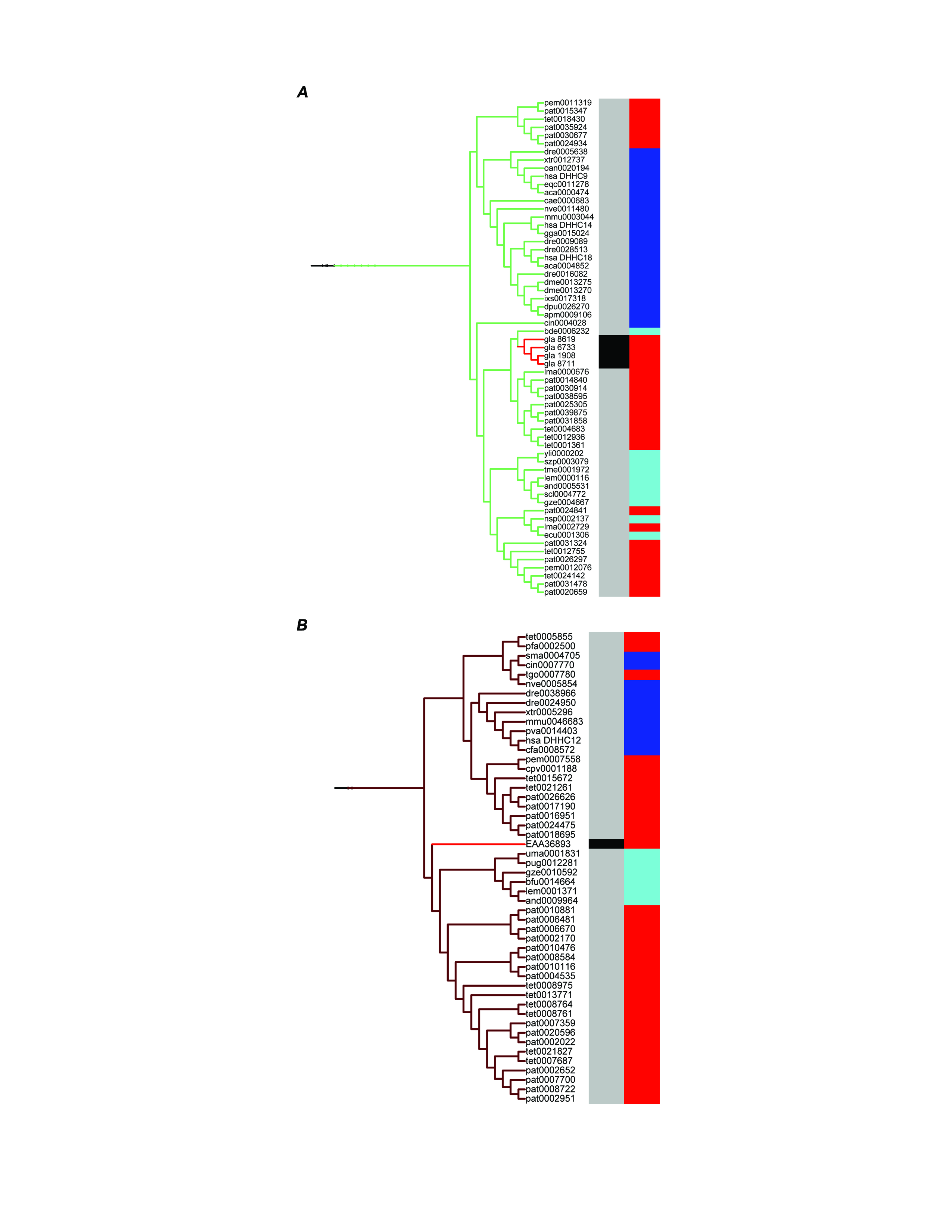

Supplement: Figure S2 — The zoomed subclade containing gla_8619, gla_6733, gla_1908, and gla_8711 (A) or EAA36893 (B) from the phylogenetic tree presented in figure 4 . Sequence taxonomic identity is displayed with colors as described in figure 4. (TIF) [file pntd.0002997.s002.tif]

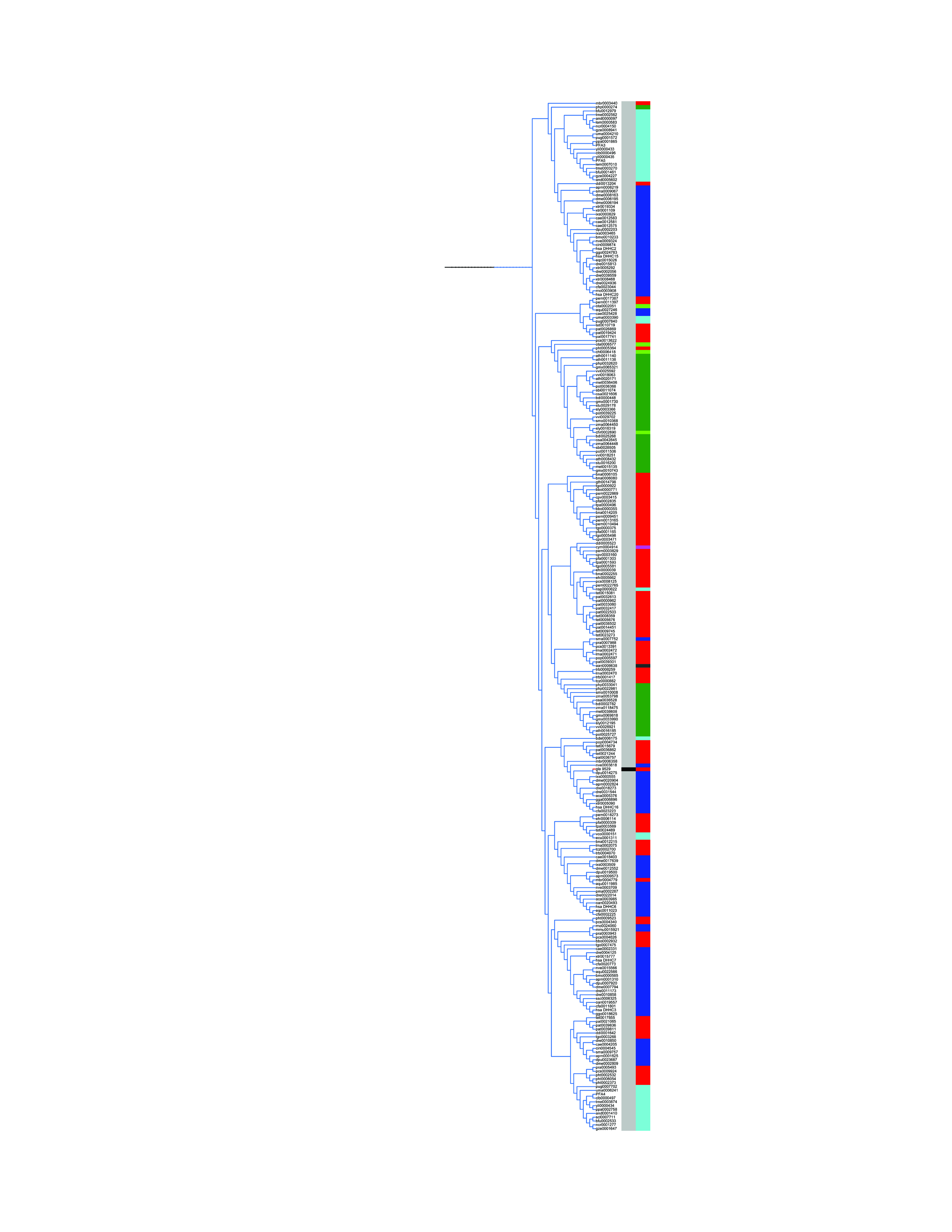

Supplement: Figure S3 — The zoomed subclade containing gla_9529 from the phylogenetic tree presented in figure 4 . Sequence taxonomic identity is displayed with colors as described in figure 4. (TIF) [file pntd.0002997.s003.tif]

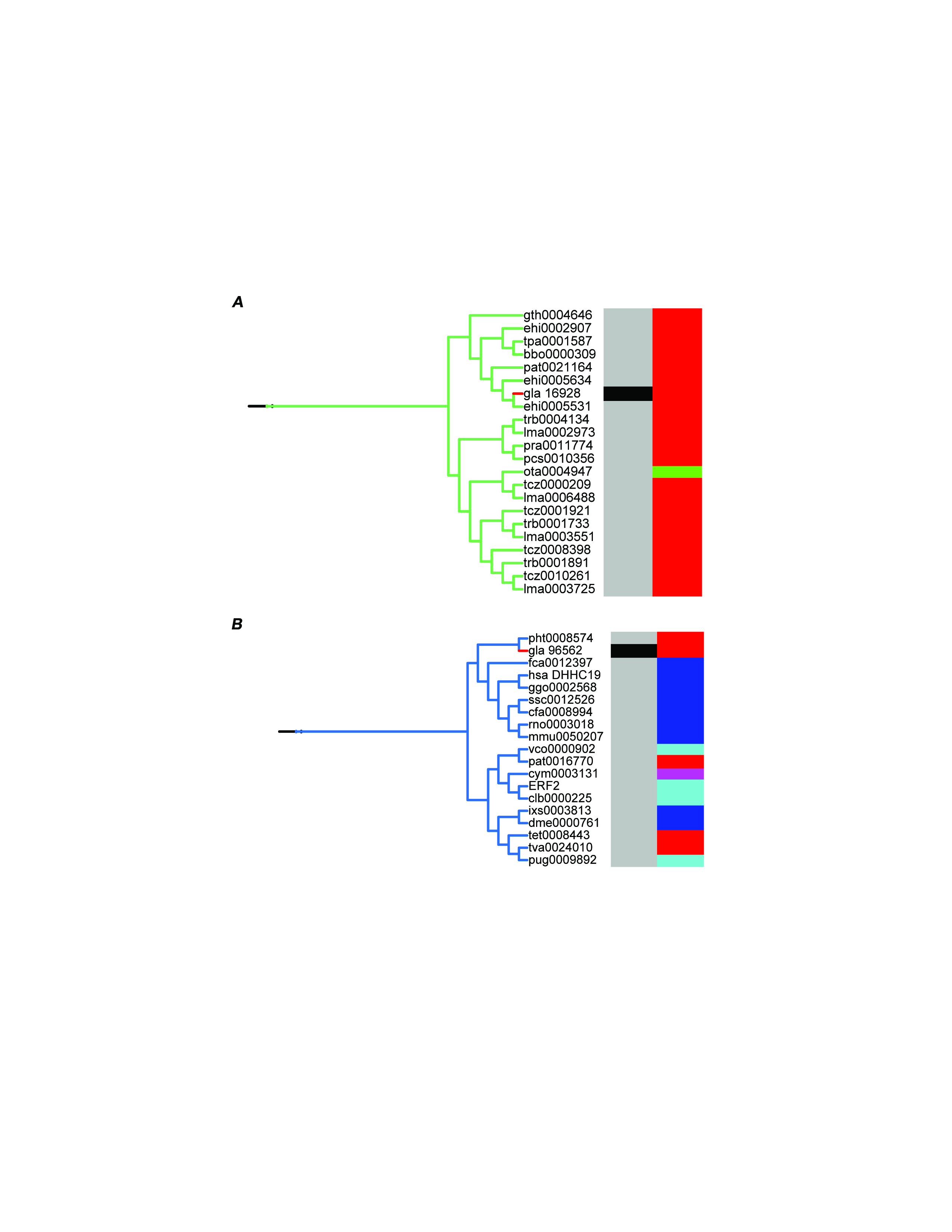

Supplement: Figure S4 — The zoomed subclade containing gla_16928 (A) or gla_96562 (B) from the phylogenetic tree presented in figure 4 . Sequence taxonomic identity is displayed with colors as described in figure 4. (TIF) [file pntd.0002997.s004.tif]

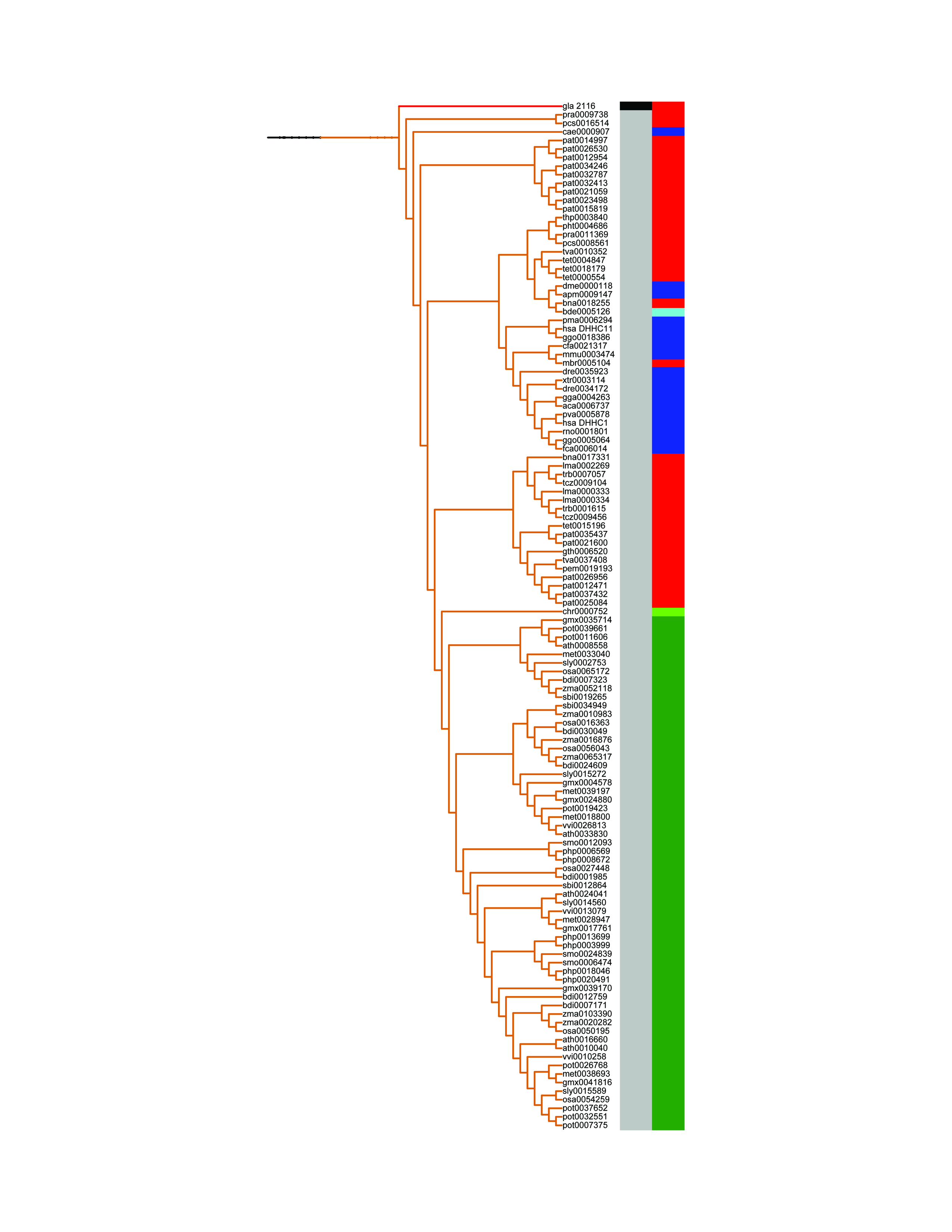

Supplement: Figure S5 — The zoomed subclade containing gla_2116 from the phylogenetic tree presented in figure 4 . Sequence taxonomic identity is displayed with colors as described in figure 4. (TIF) [file pntd.0002997.s005.tif]

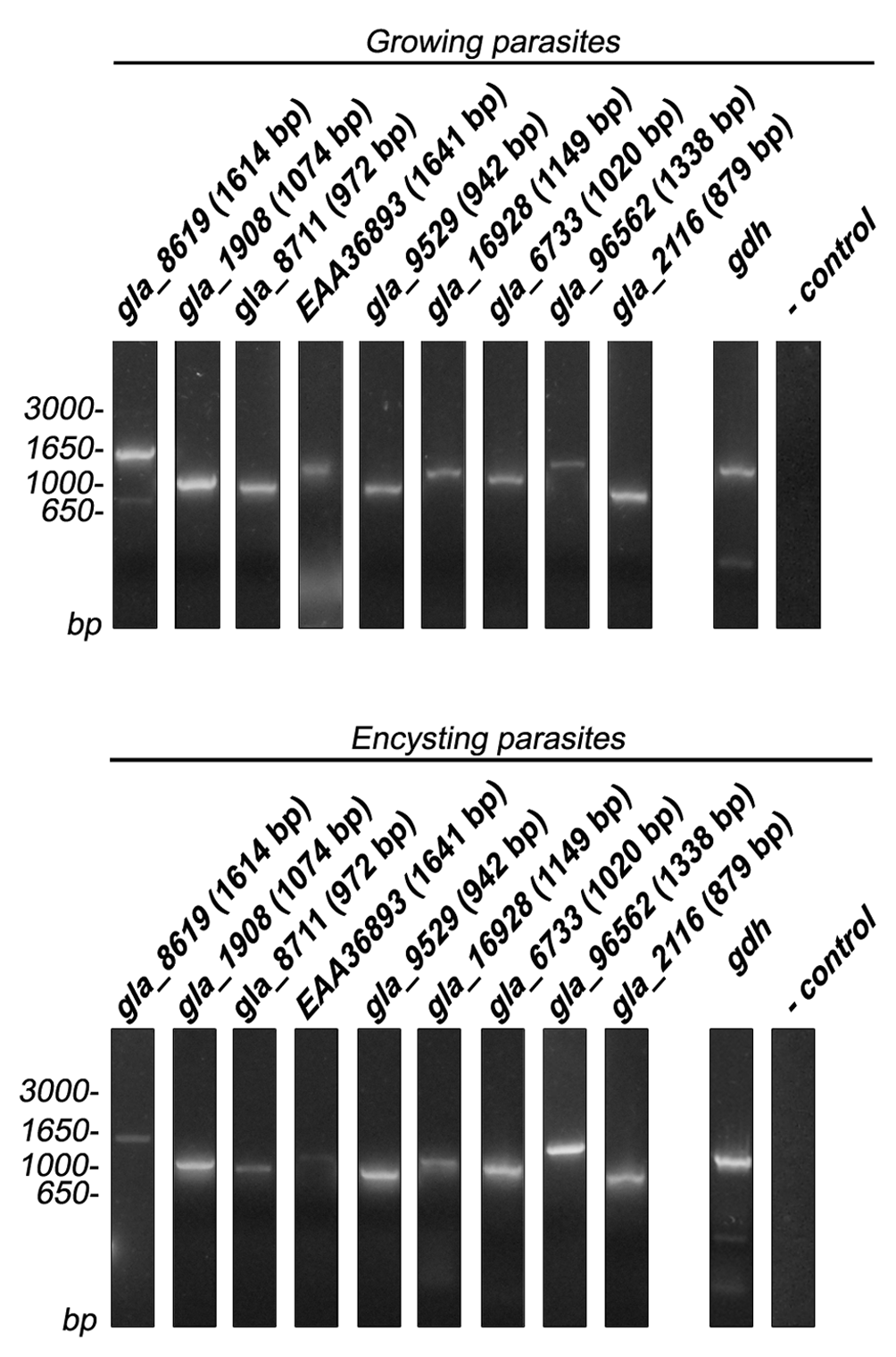

Supplement: Figure S6 — Differential expressions of Giardia dhhc genes in trophozoites and encysting parasites by semiquantitative RT-PCR. Expression of gla_8619, gla_1908, gla_8711, EAA36893, gla_9529, gla_16928, gla_6733, gla_96562, gla_2116 transcripts from growing parasites (upper panel) and 48 h encysting parasites (lower panel). Expression of glutamate dehydrogenase (gdh) mRNA fragment was tested as positive control. Expected sizes are indicated in brackets. Relative molecular weights of standards (bp) are indicated on the left. (TIF) [file pntd.0002997.s006.tif]
