# Supplementary material for: Identification of Giardia lamblia DHHC Proteins and the Role of Protein S-palmitoylation in the Encystation Process
Source: PLoS Negl Trop Dis. 2014 Jul 24;8(7):e2997. doi: 10.1371/journal.pntd.0002997 (PMC4109852; doi:10.1371/journal.pntd.0002997)
Supplement: Table S1 — Oligonucleotide primers used for Giardia DHHC cloning. (DOCX) [file pntd.0002997.s007.docx]

**Table S1. Oligonucleotide primers used for *Giardia* DHHC cloning.**

| **ORF** | **Oligonucleotide primers for DHHC protein cloning** | |
| --- | --- | --- |
|  | **Forward** | **Reverse** |
| GL50803_1908 | 5´CATTCCATGGGTAAATTCCACGGTATTGGTCGG 3´ (*NcoI*) | 5´CATTCCATGGCACAAGCGGGTCACTGGAGCATAGCAC 3´  (*EcoRV*) |
| GL50803_2116 | 5´CATTCCATGGTGAGTAAAGATGCAGTATTATCT 3´ (*NcoI*) | 5´CATTGATATCTTTTCTGAATCTATCTGCAAGGCT 3´ (*EcoRV*) |
| GL50803_16928 | 5´GTTCCATGGAAGTCCCCGATGGAAATAGT 3´ (*NcoI*) | 5´GATGTTAACTTCGTGATCCGTTGATTGCATCGG 3´ (*HpaI*) |
| GL50803_8711 | 5´CATTCCATGGTAAAGAATGGTAGTAAAATGCTTC 3´ (*NcoI*) | 5´CATTGATATCTAATTGGCTTGTTCCGCAGAGAAC 3´ (*EcoRV*) |
