# Supplementary material for: Identification of Giardia lamblia DHHC Proteins and the Role of Protein S-palmitoylation in the Encystation Process
Source: PLoS Negl Trop Dis. 2014 Jul 24;8(7):e2997. doi: 10.1371/journal.pntd.0002997 (PMC4109852; doi:10.1371/journal.pntd.0002997)
Supplement: Table S2 — Oligonucleotide primers used for semiquantitative RT-PCR. (DOCX) [file pntd.0002997.s008.docx]

**Table S2. Oligonucleotide primers used for Semiquantitative RT-PCR.**

| **ORF** | **Oligonucleotide primers for Semiquantitative RT-PCR** | |
| --- | --- | --- |
|  | **Forward** | **Reverse** |
| GL50803_16928 | 5’GTTCCATGGAAGTCCCCGATGGAAATAGT 3’ | 5’GATGTTAACTTCGTGATCCGTTGATTGCATCGG 3’ |
| GL50803_96562 | 5’CATTCCATGGCCATATTGGTCTTTCTAGTCATA 3’ | 5’CATTCAATTGTACGATGTTGCGCTTAAACTCCAA 3’ |
| GL50803_1908 | 5’CATTCCATGGGTAAATTCCACGGTATTGGTCGG 3’ | 5’CATTCCATGGCACAAGCGGGTCACTGGAGCATAGCAC 3’ |
| GL50803_2116 | 5’CATTCCATGGTGAGTAAAGATGCAGTATTATCT 3’ | 5’CATTGATATCTTTTCTGAATCTATCTGCAAGGCT 3’ |
| GL50803_6733 | 5’CATTGGCGCGCCCTGTCGCGAGCCTCGTTTGTG 3’ | 5’CATTGCGGCCGCTTAGATATCTTCGCGTGTTAGCGC 3’ |
| GL50803_8619 | 5’CATTGGGCCCCTAATTAAGCTATCCAAAGACATC 3’ | 5’CATTCCCGGGGTGTGCAACCTCGTTCATTTCACG 3’ |
| GL50803_8711 | 5’CATTCCATGGTAAAGAATGGTAGTAAAATGCTTC 3’ | 5’CATTGATATCTAATTGGCTTGTTCCGCAGAGAAC 3’ |
| GL50803_9529 | 5’GTTCCATGGGCAAGGAGGGCCCCAATAGG 3’ | 5’GATGATATCATTCATCTCTTCACCCGATAAATC 3’ |
| EAA36893 | 5’GTTGGATCCATGGTCAGTTGTGTCGACAAGATCTTC 3’ | 5’GTATGCATGCATTTTCCAGACCCTGGACAATTCTATC 3’ |
| GDH  (GL50803_21942) | 5’CACCATGCCTGCCCAGACGATCGAGGAG 3’ | 5’TCACACGCAGCCCTGCTCGATCATCAT 3’ |
