# Supplementary material for: Identification of Giardia lamblia DHHC Proteins and the Role of Protein S-palmitoylation in the Encystation Process
Source: PLoS Negl Trop Dis. 2014 Jul 24;8(7):e2997. doi: 10.1371/journal.pntd.0002997 (PMC4109852; doi:10.1371/journal.pntd.0002997)
Supplement: Table S3 — Oligonucleotide primers used for qRT-PCR. (DOCX) [file pntd.0002997.s009.docx]

**Table S3. Oligonucleotide primers used for qRT-PCR.**

| **ORF** | **Oligonucleotide primers for qRT-PCR** | |
| --- | --- | --- |
|  | **Forward** | **Reverse** |
| GL50803_16928 | 5’TCGTCCTGGGCTCCTCTTG 3’ | 5’ TGCTTTCCCCTTTTCCTTGA 3’ |
| GL50803_96562 | 5’CGACCGCCTCGTTCTCAT 3’ | 5’GATCAAAGTCCAGCACACACCTT 3’ |
| GL50803_1908 | 5’GGTGGCGGCGTGTCTTT 3’ | 5’CCTGGGCCGACAGGTCTA 3’ |
| GL50803_2116 | 5’GCACCGTTTTCGGCTACAAG 3’ | 5’GTGGGCTGGCTCGCATAG 3’ |
| GL50803_6733 | 5’GCAAAACTCAAGAAAACGCAAA 3’ | 5’CGTTGCTTGAATGCCTGGAT 3’ |
| GL50803_8619 | 5’TTCCAACCCAAACACCTCAAA 3’ | 5’CACGCGTCGACAATCTCTTG 3’ |
| GL50803_8711 | 5’CCCGCCCGCAGTTACA 3’ | 5’CAGTCCAGACAGAAGCGTACGT 3’ |
| GL50803_9529 | 5’CGGAAAATGCCACAATGCT 3’ | 5’CCGCAGATGCTGCAGTGA 3’ |
| EAA36893 | 5’TGCAATCGATGCGTCCTTAA 3’ | 5’CAACGCAGTTCCCAATCCA 3’ |
| CWP1 (GL50803_5638) | 5’AACGCTCTCACAGGCTCCAT 3’ | 5’AGGTGGAGCTCCTTGAGAAATTG 3’ |
| CWP2 (GL50803_5435) | 5’TAGGCTGCTTCCCACTTTTGAG 3’ | 5’CGGGCCCGCAAGGT 3’ |
| CWP3 (GL50803_2421) | 5’GCAAATTGGATGCCAAACAA 3’ | 5’GACTCCGATCCAGTCGCAGTA 3’ |
| GDH (GL50803_21942) | 5’AGGGCGGCTCCGACTTT 3’ | 5’AGCGCATGACCTCGTTGTC 3’ |
